# *Pseudomonas aeruginosa* ExoU augments neutrophil transepithelial migration

**DOI:** 10.1371/journal.ppat.1006548

**Published:** 2017-08-03

**Authors:** Michael A. Pazos, Bernard B. Lanter, Lael M. Yonker, Alex D. Eaton, Waheed Pirzai, Karsten Gronert, Joseph V. Bonventre, Bryan P. Hurley

**Affiliations:** 1 Mucosal Immunology & Biology Research Center, Massachusetts General Hospital for Children, Boston, Massachusetts, United States of America; 2 Pediatrics, Harvard Medical School, Boston, Massachusetts, United States of America; 3 Vision Science Program, School of Optometry, University of California at Berkeley, Berkeley, California, United States of America; 4 Renal Division and Biomedical Engineering Division, Brigham and Women's Hospital, Department of Medicine, Harvard Medical School, Boston, Massachusetts, United States of America; University of Washington, UNITED STATES

## Abstract

Excessive neutrophil infiltration of the lungs is a common contributor to immune-related pathology in many pulmonary disease states. In response to pathogenic infection, airway epithelial cells produce hepoxilin A_3_ (HXA_3_), initiating neutrophil transepithelial migration. Migrated neutrophils amplify this recruitment by producing a secondary gradient of leukotriene B_4_ (LTB_4_). We sought to determine whether this two-step eicosanoid chemoattractant mechanism could be exploited by the pathogen *Pseudomonas aeruginosa*. ExoU, a *P*. *aeruginosa* cytotoxin, exhibits phospholipase A2 (PLA2) activity in eukaryotic hosts, an enzyme critical for generation of certain eicosanoids. Using in vitro and in vivo models of neutrophil transepithelial migration, we evaluated the impact of ExoU expression on eicosanoid generation and function. We conclude that ExoU, by virtue of its PLA2 activity, augments and compensates for endogenous host neutrophil cPLA2α function, leading to enhanced transepithelial migration. This suggests that ExoU expression in *P*. *aeruginosa* can circumvent immune regulation at key signaling checkpoints in the neutrophil, resulting in exacerbated neutrophil recruitment.

## Author summary

*Pseudomonas aeruginosa* is an opportunistic pathogen that causes acute pneumonia in immune compromised patients, and infects 70–80% of patients suffering from cystic fibrosis. Infections can result in excessive airway inflammation, which lead to immune-mediated lung damage, in particular through the action of recruited white blood cells known as neutrophils. Certain strains of *P*. *aeruginosa* produce the exotoxin ExoU, which has been associated with increased virulence. ExoU causes host cell lysis by hydrolyzing host membrane lipids through its phospholipase activity. However, host phospholipases play a key role in immune signaling by mediating the production of lipids known as eicosanoids. We investigated whether separate from its cytolytic activity, ExoU could modulate host immune responses through its phospholipase activity by hijacking eicosanoid production. Using *in vitro* and *in vivo* models of neutrophil recruitment, we find that ExoU producing strains of *P*. *aeruginosa* elicit higher levels of the eicosanoid chemoattractant leukotriene B4 from migrated neutrophils. This results in increased neutrophil transepithelial migration. This work reveals a new mechanism for how bacterial pathogens alter our immune function, and highlights a new potential therapeutic strategy for moderating *Pseudomonas* pathogenesis in patients with cystic fibrosis and acute pneumonia.

## Introduction

Neutrophil infiltration of the airways is the pathological hallmark of pulmonary inflammatory disorders such as acute pneumonia and cystic fibrosis (CF) [1–3]. The most common pathogen associated with CF is *Pseudomonas aeruginosa* [4], an opportunistic pathogen infecting 70–80% of CF patients by the time they reach their teens [5]. This microbial infection exacerbates inflammation, leading to persistent neutrophil accumulation and subsequent tissue destruction. A finer mechanistic understanding of neutrophil trafficking could provide useful targets for limiting pathophysiology in CF [6]. Neutrophil transit from the periphery to the airspace involves a coordinated series of signaling events involving endothelial adhesion, diapedesis, and navigation through the interstitium before crossing the airway epithelium. As the final step prior to reaching the airway, transepithelial migration is an important therapeutic target for restraining neutrophilic damage of the airway without broad immune suppression [7].

Many chemotactic signals are required to maintain successful neutrophil navigation from the periphery to the lumen [8], but a unique requirement in mediating transepithelial recruitment is the eicosanoid chemotactic axis of hepoxilin A_3_ (HXA_3_) and leukotriene B_4_ (LTB_4_). HXA_3_ is an apically directed, epithelial-derived chemoattractant shown to initiate pathogen-induced neutrophil transepithelial migration to a variety of bacterial triggers [9,10], including infection with *P*. *aeruginosa* [11]. Following initial migration to HXA_3_ chemotactic gradients, neutrophils extend the basolateral-to-apical chemotactic gradient by producing LTB_4_, thereby amplifying the magnitude of neutrophil recruitment [12]. HXA_3_ and LTB_4_ are derived from the enzymatic oxidation of arachidonic acid (AA) by 12-lipoxygenase (12-LOX) and 5-lipoxygenase (5-LOX), respectively. AA availability is tightly regulated by members of the phospholipase A2 (PLA2) family of enzymes, and is the rate-limiting step in the production of eicosanoids. The PLA2 enzyme family has over 20 members segregated into several major classes with distinct structure, function, and selective eicosanoid-generating capacity [13,14]. cPLA2α is closely associated with inflammatory eicosanoid generation and is critical for LTB_4_ production [15], while HXA_3_ production requires a distinct, currently unknown PLA2 family member [16].

In addition to mammalian PLA2 enzymes, many bacterial pathogens express enzymes with PLA2 activity [17–20]. ExoU is one such bacterial PLA2 produced by some strains of *P*. *aeruginosa* [5,19,21,22]. ExoU is injected into host cytoplasm via the type III secretion system, where it is activated by ubiquitin [23,24] and localized to the plasma membrane [25]. ExoU exhibits phospholipase activity due to the presence of a patatin domain which displays functional similarities to mammalian cPLA2 and iPLA2 enzymes [26,27]. ExoU has been widely described as a potent cytotoxin, rapidly killing a number of host cells including epithelial cells and neutrophils [26,28–30]. In addition to the cytotoxic effects of ExoU, the toxin’s PLA2 activity is associated with increased eicosanoid production, in particular prostaglandin E_2_ (PGE_2_) [31,32].

Several recent reports suggest that *P*. *aeruginosa* may benefit from promoting inflammation in the airway [3,33–35]. We considered whether, distinct to its cytotoxic functions, ExoU serves to modulate the production of eicosanoid chemoattractants and promote neutrophil recruitment to the infected airway. Using both *in vitro* and *in vivo* models of neutrophil trafficking, we demonstrate that ExoU expression is capable of augmenting selective eicosanoid production in both epithelial cells and neutrophils. In neutrophils, this eicosanoid generation contributes to chemotactic signaling and recruitment of additional neutrophils to the pulmonary lumen independently of its cytotoxic effects. Taken together, we observe that ExoU exploits endogenous mechanisms of neutrophil trafficking by increasing neutrophil-derived LTB_4_, resulting in increased recruitment across infected pulmonary epithelium.

## Results

### ExoU-associated PLA2 activity selectively enhances production of eicosanoids in epithelial cells

We evaluated whether ExoU-associated PLA2 activity was observable in our epithelial cell line models in the context of minimal ExoU-mediated cellular cytotoxicity. Infection of A549 lung epithelial cells with the ExoU-expressing PA14 strain of *P*. *aeruginosa* was associated with an increase in cytotoxicity (S1 Fig). We did not observe *P*. *aeruginosa*-induced A549 cytotoxicity when infecting with PAO1, a strain that naturally lacks the *exoU* gene, or PA14 ΔExoU, a strain with an insertional mutation of the *exoU* gene in the PA14 background. By limiting the infectious dose of the PA14 strain we reduced the cytotoxic impact to background levels for the duration of our 1h infection and subsequent incubation (S1 Fig). Using the lower dose that elicits no cytotoxicity, we explored whether the PLA2 activity of ExoU could augment host eicosanoid synthesis.

**Fig 1:**
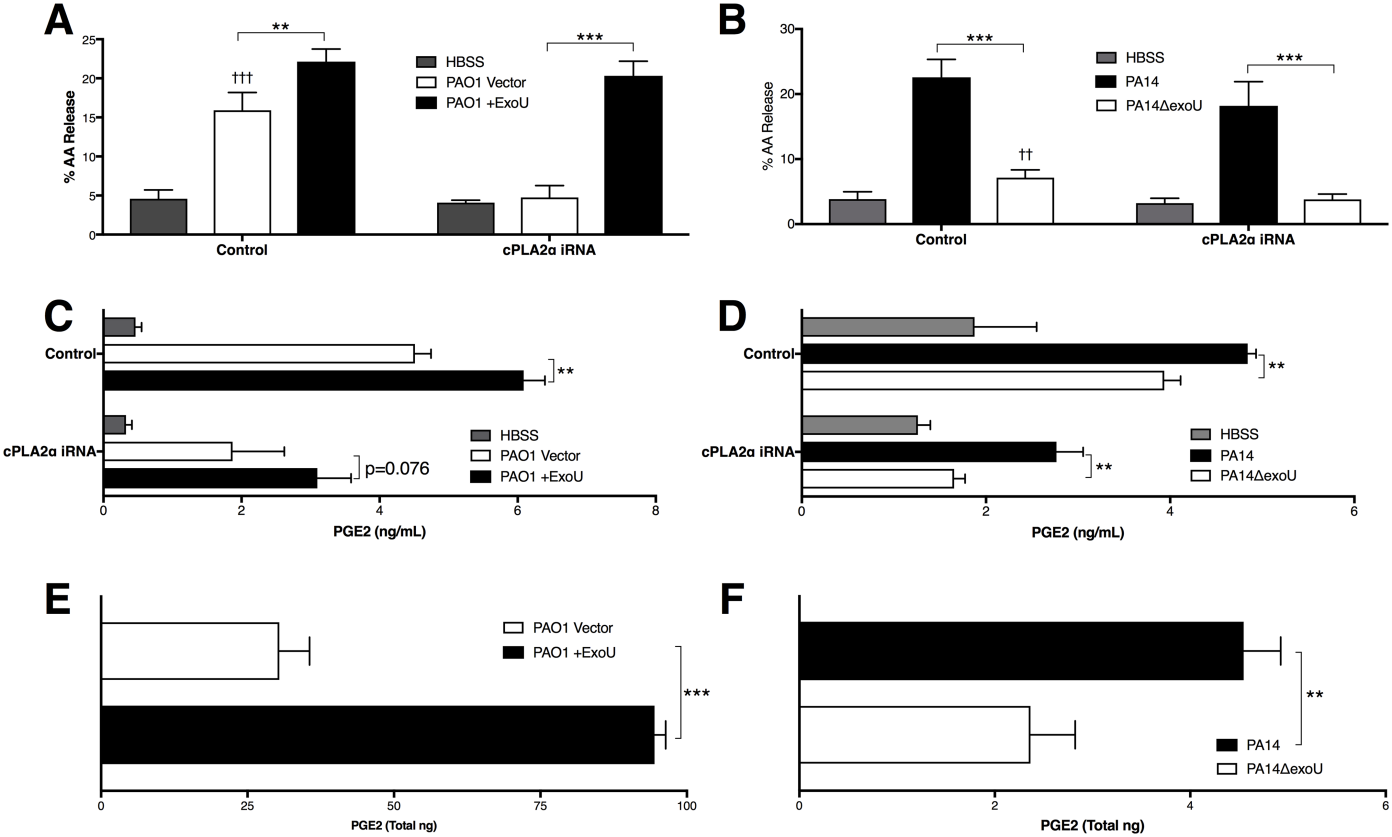
ExoU-associated PLA2 activity enhances production of epithelial arachidonic acid and PGE_2_. Lung epithelial monolayers were infected with matched strains of PAO1 or PA14 that either express or lack ExoU. (A, B) Arachidonic acid release was assessed following infection of confluent 24w plate-grown A549 cells with ExoU-expressing or -lacking strains of *P*. *aeruginosa*. Control cells stably express irrelevant control RNAi, while cPLA2α iRNA cells express inhibitory RNA targeting *cpla2α* mRNA. (C, D) Total PGE_2_ production was measured in the supernatant of infected A549 cells by immunoassay following 1h infection with the indicated strain, and a subsequent 2h incubation following epithelial wash. Total PGE_2_ (E, F) levels were measured in lipid-extracted supernatants by LC/MS/MS following infection of non-transfected H292 lung epithelial cells. Data are represented as means +/- SD and are representative of multiple independent experiments. **p < 0.01, ***p < 0.001, indicate comparison between ExoU lacking or expressing genetically matched strains. n.s. indicates a non-statistically significant difference. †† p <0.01, ††† p <0.001 indicate comparison to HBSS control.

We previously demonstrated that infection of epithelial cell lines with *P*. *aeruginosa* activates the host PLA2 isoform cPLA2α independently of ExoU expression, leading to enhanced AA liberation and synthesis of the cyclooxygenase-derived eicosanoid PGE_2_ [16]. We therefore analyzed liberation of AA from lung epithelial cells in response to infection with either ExoU-expressing or non-expressing strains of *P*. *aeruginosa* to determine whether enhanced PLA2 activity is associated with ExoU expression. PAO1 is a strain of *P*. *aeruginosa* that does not naturally express ExoU. We utilized a strain transformed to express ExoU and its chaperone SpcU (PAO1 +ExoU), as well as a strain transformed with a non-expressing plasmid vector (PAO1 Vector) [21]. In addition to manipulation of *P*. *aeruginosa* ExoU expression, we employed stably transfected A549 cell lines that either express minimal cPLA2α due to the presence of an RNAi plasmid targeting cpla2α-mRNA, or a plasmid control exhibiting normal cPLA2α expression (S2 Fig) [16]. Infection of the control A549 cells with either PAO1 strain resulted in a significant increase in AA release, consistent with previous studies (Fig 1A) [16]. Interestingly, ExoU expression was associated with a further 39% increase in AA release. To evaluate natural ExoU expression, we utilized the ExoU-expressing strain PA14 and PA14ΔexoU. Infection of control A549 cells with either strain again resulted in a significant increase in AA release, with ExoU expression in PA14 being associated with significantly more AA liberation (Fig 1B). Infection of cPLA2α-suppressed cells exhibited blunted AA release in response to infection in the absence of ExoU expression (Fig 1A and 1B). In contrast, when infected with strains expressing ExoU, cPLA2α-suppressed epithelial cells remain capable of inducing significant AA release.

PGE_2_ generation in response to *P*. *aeruginosa* infection relies on cPLA2α-mediated AA liberation [16], and ExoU-expression in *P*. *aeruginosa* has been associated with further increased production of PGE_2_ in multiple cell lines [31,32]. We observed that PGE_2_ production by A549 cells displayed a similar pattern to AA release. ExoU expression was associated with significant increases in PGE_2_ production in control A549 cells in both PAO1 (Fig 1C) and PA14 (Fig 1D) strains, and strongly associated with PGE_2_ release in cPLA2α-suppressed cells. We also tested whether ExoU expression was associated with increased infection-induced production of PGE_2_ in additional epithelial cell lines. Supernatants were collected from infected H292 human epithelial cells and analyzed for PGE_2_ content by LC/MS/MS. Infection with PAO1 +ExoU (Fig 1E) and PA14 (Fig 1F) was associated with a significant increase in PGE_2_ production compared to infection with their ExoU-negative controls. These data support existing literature that ExoU functions as a PLA2 enzyme in mammalian cells capable of directly participating in the generation of eicosanoids in a setting where ExoU-mediated cytotoxicity is not observed.

We then investigated whether the eicosanoid-generating capacity of ExoU, in conjunction with endogenous lipoxygenases, could contribute to the production of epithelial-derived neutrophil chemoattractants. We previously demonstrated that endogenous mammalian cPLA2α isoform was dispensable for *P*. *aeruginosa*-induced HXA_3_ generation, although PLA2 activity is required [16]. Because the degree of functional overlap between endogenous PLA2 enzymes and ExoU is unclear, we sought to determine whether infection with ExoU-expressing strains of *P*. *aeruginosa* resulted in significant differences in epithelial HXA_3_ production. H292 epithelial monolayers were infected with paired strains of PAO1 and PA14 expressing or lacking ExoU. Lipid-extracted supernatants were analyzed by LC/MS/MS for production of HXA_3_. We were unable to detect any significant differences in total production of HXA_3_ (S3A and S3B Fig). In order to further examine whether ExoU expression in infection strains resulted in functional differences in epithelial-derived lipid-associated chemotactic bioactivity, we used an established assay to quantify HXA_3_-associated bioactivity [12,36]. Flasks of A549 cells were infected with PAO1 vector, PAO1 +ExoU, PA14 or PA14ΔexoU to stimulate HXA_3_ synthesis. Lipid-extracted supernatant was collected, serially diluted, and used as a chemotactic gradient in a subsequent neutrophil migration assay. The number of neutrophils migrating across H292 monolayers in response to a gradient of lipids derived from A549 cells represents HXA_3_-associated bioactivity and is depicted as a % of Control. Control is set at 100% and represents the magnitude of the migratory response to undiluted lipids derived from A549 cells infected with either parental bacterial strain PAO1-Vector (S3C Fig) or PA14 (S3D Fig) and all other conditions are depicted as a relative percentage to the measured response of Control. We did not observe any significant difference between epithelial-derived chemotactic activity associated with ExoU expression based on the magnitude of the migratory response (S3C and S3D Fig), consistent with the findings from LC/MS/MS analysis demonstrating the lack of a difference in HXA_3_ release following infection with strains that either lack or express ExoU (S3A and S3B Fig). Taken together, these data indicate that ExoU exhibits PLA2 activity in epithelial cells by liberating AA, leading to enhanced generation of PGE_2_. ExoU, however, does not appear to significantly impact epithelial HXA_3_ generation, despite the availability of AA, a shared precursor molecule for both HXA_3_ and PGE_2_.

### ExoU expression is associated with increased neutrophil transepithelial migration and increased LTB_4_ generation

Although we were unable to observe a significant impact of ExoU on epithelial-derived lipid neutrophil chemoattractants, we considered whether ExoU may contribute to chemotactic eicosanoid production in migrating neutrophils. Following their response to HXA_3_, migrated neutrophils amplify the neutrophil migratory response by producing LTB_4_ [12]. Although endogenous cPLA2α is dispensable for epithelial HXA_3_ production [13,16], it plays a critical role in production of LTB_4_ in certain contexts [15]. The relative ExoU expression in our bacterial strains was determined by preparing lysates and probing for the presence of ExoU via western blot. The artificially expressing ExoU strain whereby ExoU is encoded on an inserted vector (PAO1 +ExoU) appears to express significantly more protein than the naturally expressing ExoU wild-type strain (PA14) and neither the PAO1 vector alone, nor the PA14ΔexoU exhibit any ExoU expression as expected (S4A Fig). Further, we quantified the difference in expression between PAO1 +ExoU and PA14 and found the former to express slightly great than 10-fold more ExoU than the later when assessed by densitometry analysis (S4B and S4C Fig). The potential impact of ExoU expression on viability of H292 lung epithelial grown on inverted transwells was also examined (S5A and S5B Fig). Polarized H292 cells grown on transwell supports were resistant to ExoU cytotoxicity during the 1h infection and subsequent incubation time. We also evaluated the cytotoxicity to neutrophils and observed minimal evidence of cytotoxicity to the neutrophils in this assay (S5C Fig). ExoU-associated cytotoxicity of neutrophils has previously been observed, though at later time points of infection [28,37].

**Fig 2:**
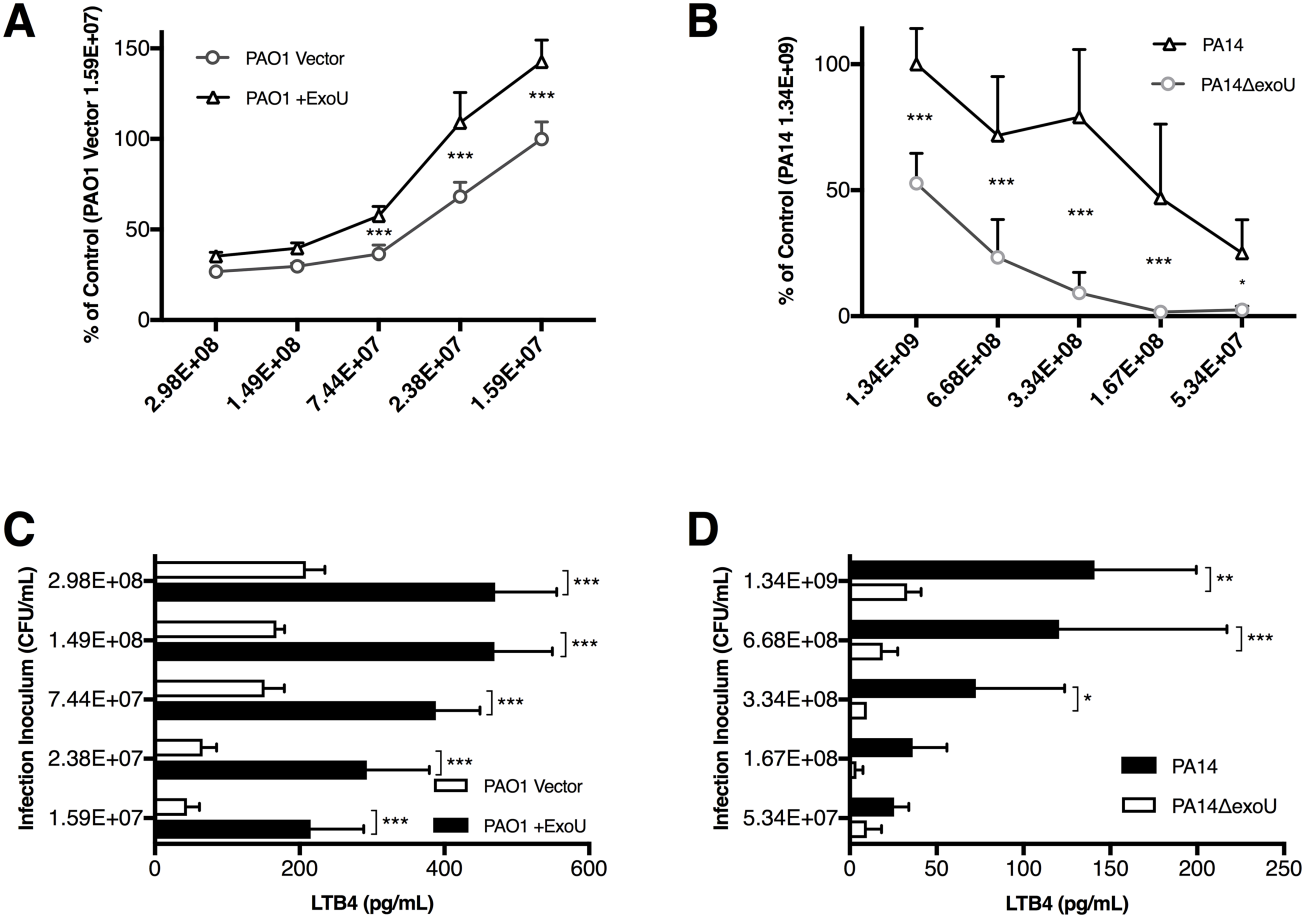
Bacterial ExoU is associated with increased neutrophil transepithelial migration and LTB_4_ generation. Inverted H292 monolayers were infected with various doses of matched strains of either (A, C) PAO1 or (B, D) PA14 that express or lack ExoU. Neutrophils were provided to the basolateral surface and allowed to migrate. Neutrophil migration was reported as a percent of Control. The magnitude of the neutrophil migration response to parental strains PAO1 Vector or PA14 at infection doses of 1.59E+07 and 1.34E+09 respectively were considered the Control and set at 100%. We assayed (A, B) total neutrophil migration, as well as (C, D) total LTB_4_ in the apical space following 2h migration. Data are shown as mean +/- SD, and are representative of multiple independent experiments. *p ≤ 0.05, **p < 0.01, ***p < 0.001 indicate comparison between ExoU lacking or expressing genetically matched strains at the same concentration.

Inverted H292 epithelial cells were infected with matched strains of *P*. *aeruginosa* at multiple doses to determine the extent to which each of these strains were capable of instigating neutrophil trans-epithelial migration. Neutrophil trans-epithelial migration was measured as a % of Control, in which the bacterial infection of either parental strain (PAO1 Vector or PA14) at an infection dose that represents the highest magnitude of migratory response (1.59E+07 or 1.34E+09 respectively) was set to 100% (Fig 2). We observed a persistent and significant increase in neutrophil migration in response to infection with PAO1 +ExoU when compared to PAO1 Vector (Fig 2A). Previous studies investigating the magnitude of the neutrophil migratory response to varying doses of PAO1 revealed that the response follows a highly reproducible bell-shaped pattern whereby high doses of infection result in minimal transepithelial migration, however, as you decrease the PAO1 infection concentration the magnitude of the PMN transepithelial migration increases and eventually plateaus after which lower PAO1 infections concentration result in lower PMN transepithelial migration until a very low PAO1 infection concentration is reached where no detectible migratory response is observed [38]. Despite PAO1 + ExoU demonstrating significantly increased neutrophil transepithelial migration at multiple infection concentrations compared to PAO1 Vector, both PAO1 Vector and PAO1 +ExoU appear to follow this intriguing pattern of response with data depicted herein representing the left half of this highly reproducible bell curve response (Fig 2A). We repeated this experiment using paired PA14 strains, and observed a similar significant increase in migration associated with ExoU expression (Fig 2B). At the infection concentrations examined for PA14 and PA14ΔexoU, a more predictable dose-dependent positive correlation between the number of infecting bacteria and magnitude of migratory response was observed, however, we cannot rule out that this may represent the right half of a bell curve relationship with even higher doses of PA14 resulting in a diminishing migratory response (Fig 2B).

Epithelial production of HXA_3_ was not significantly impacted by ExoU expression (S3 Fig) as a possible explanation for the enhanced migratory response associated with ExoU, thus we next evaluated total LTB_4_ production in the apical space following migration of neutrophils. In both strain pairs, LTB_4_ generation was strongly correlated with the presence of ExoU and neutrophil migration (Fig 2C and 2D). These data indicate that while ExoU does not modulate epithelial production of HXA_3_, it does, however, significantly impact neutrophil transepithelial recruitment in this transwell model system, possibly by influencing neutrophil production of LTB_4_ during the migration process.

### ExoU circumvents cPLA2α blockade in neutrophils and rescues blunted neutrophil transepithelial migration

cPLA2α function is tightly regulated by expression, cellular localization, as well as phosphorylation by the MAPK/ERK pathway [15]. Inhibition of ERK activity reduces *P*. *aeruginosa*-induced AA liberation through blockade of cPLA2α activity [16]. In contrast, expression of ExoU can augment AA liberation even in the context of cPLA2α suppression (Fig 1). Since ExoU is associated with increased neutrophil transepithelial migration and increased apical LTB_4_ concentrations, we sought to determine if the presence of ExoU impacts *P*. *aeruginosa*-induced migration by directing neutrophil synthesis of LTB_4_ independently of host cPLA2α. To properly address this question, we investigated whether pharmacological inhibition of neutrophil cPLA2α activation serves to prevent LTB_4_ synthesis, and whether the presence of ExoU could override such a blockade.

**Fig 3:**
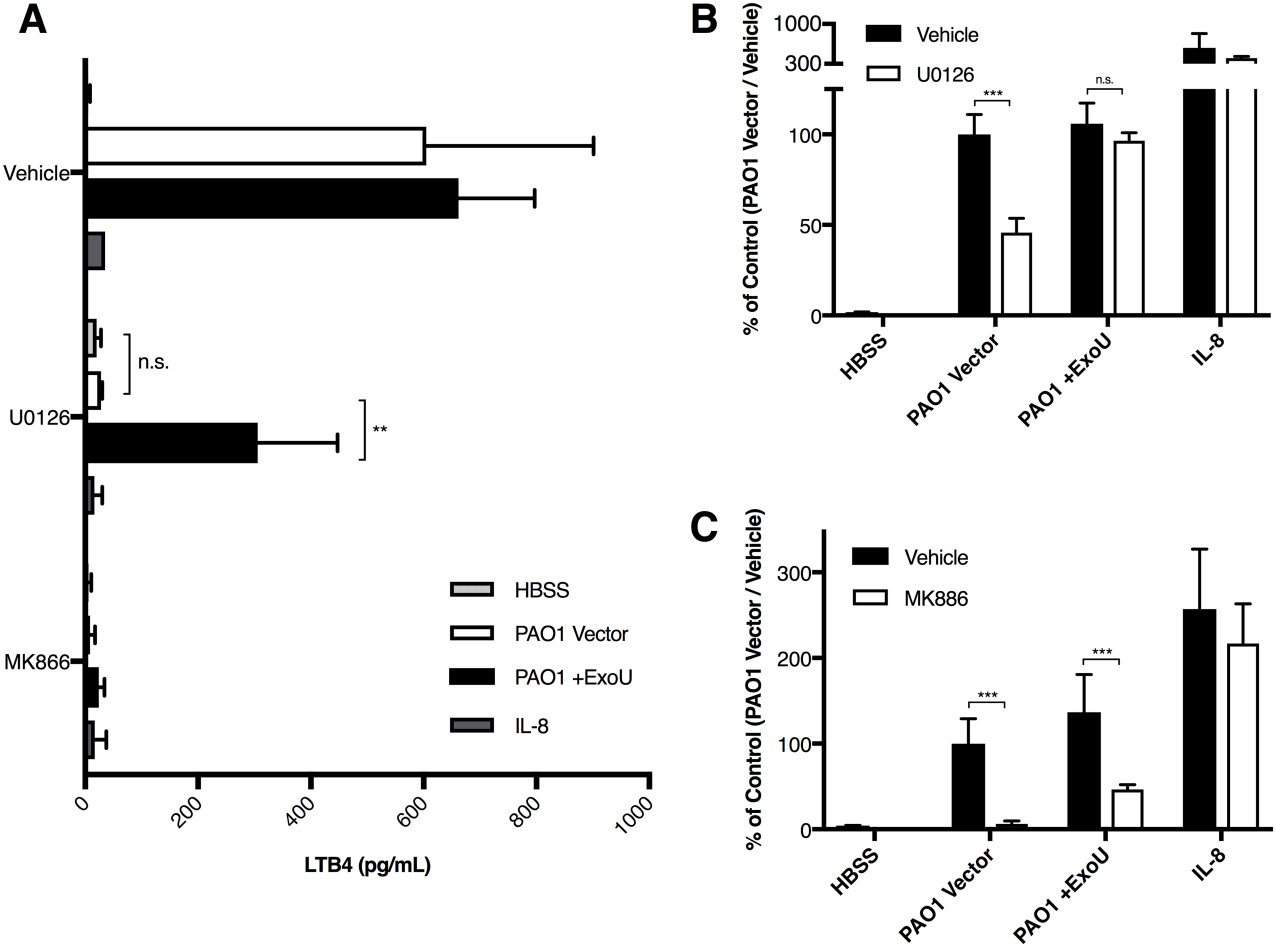
ExoU circumvents suppression of cPLA2α activity. Neutrophils were treated with pharmacological inhibitors of the LTB_4_ biosynthetic pathway U0126 (20μM), and MK886 (2μM) for 1h prior to migration, and allowed to migrate across untreated H292 epithelial transwell monolayers in response to infection or exogenous gradients of IL-8. (A) Apical supernatant was sampled following migration and assayed for total LTB_4_ by immunoassay. Migration of neutrophils treated with (B) U0126, (C) MK886, or their respective vehicle controls was assessed by myeloperoxidase assay. Neutrophil migration was measured as % of Control, in which the migration response of vehicle treated neutrophils to either parental strain (PAO1 Vector or PA14) were set to 100%. Data are shown as mean +/- SD, and are representative of multiple independent experiments. **p < 0.01, ***p < 0.001 between the indicated conditions. n.s. indicates a non-statistically significant difference.

Neutrophils were pretreated with the ERK inhibitor U0126 to inhibit endogenous cPLA2α activation. Following migration of vehicle-treated neutrophils, we observed robust LTB_4_ release into the apical space in response to infection with either PAO1 Vector or PAO1 +ExoU (Fig 3A). U0126-treated neutrophils migrating in response to epithelial infection with PAO1 +ExoU released significant amounts of LTB_4_ into the apical supernatant, while the U0126-treated neutrophils migrating in response to epithelial infection with PAO1 Vector released only background levels of LTB_4_ (Fig 3A). Neutrophils were also pretreated with the 5-lipoxygenase-activating protein (FLAP) inhibitor MK886 to target LTB_4_ production downstream of cPLA2α function. Only background levels of LTB_4_ were detected following migration of FLAP-inhibited neutrophils in response to infection with either PAO1 Vector or PAO1 +ExoU (Fig 3A).

The total migration of neutrophils correlated strongly with altered LTB_4_ generation following pharmacological inhibition. U0126-treated neutrophils migrated poorly in response to epithelial infection with PAO1 Vector compared to vehicle control-treated neutrophils (Fig 3B). Migration in response to epithelial infection with PAO1 +ExoU, on the other hand, was equally robust irrespective of ERK inhibition in the neutrophil. Migration of MK886-treated neutrophils was significantly reduced in response to epithelial infection with *P*. *aeruginosa*, irrespective of ExoU expression (Fig 3C). Migration to exogenous IL-8 gradients, which does not rely on the generation of LTB_4_ by migrating neutrophils, was not impacted by drug treatment with either U0126 or MK886 (Fig 3).

These data suggest that ExoU can functionally overcome inhibition of cPLA2α-mediated arachidonic acid liberation in neutrophils, which results in LTB_4_ generation. ExoU-associated production of LTB_4_ was sufficient to restore neutrophil migration in the context of cPLA2α inhibition, whereas inhibition of 5-LOX, the synthetic enzyme located downstream of both cPLA2α and ExoU, resulted in significant impairments in LTB_4_ generation and neutrophil recruitment, irrespective of the presence of ExoU.

### ExoU compensates for absence of cPLA2α, but not 5-LOX, in a mouse *in vitro* model of neutrophil transepithelial migration

To evaluate the hypothesis that ExoU can compensate for cPLA2α deficiency in the generation of LTB4 without the potential off-target impact of pharmacological inhibition, bone marrow neutrophils were collected from mice with a targeted deletion in cpla2α for use in a murine co-culture migration model system [12]. Mouse lung epithelial cells (MLE12) were grown inverted on transwell filters and infected with PAO1 Vector or PAO1 +ExoU. We first evaluated the impact of ExoU expression on the viability of MLE12 lung epithelial cells cultured on inverted transwells (S6A and S6B Fig). Polarized MLE12 cells grown on transwell supports were resistant to ExoU cytotoxicity during the 1h infection and subsequent incubation time. We additionally evaluated the potential for ExoU-mediated cytotoxicity of neutrophils and we did not observe significant ExoU-associated cell death (S6C Fig). Whole bone marrow was collected from mice then supplied to the basolateral compartment of the transwell. Bone marrow neutrophils selectively migrate in response to epithelial infection or imposed exogenous chemotactic gradients [12].

**Fig 4:**
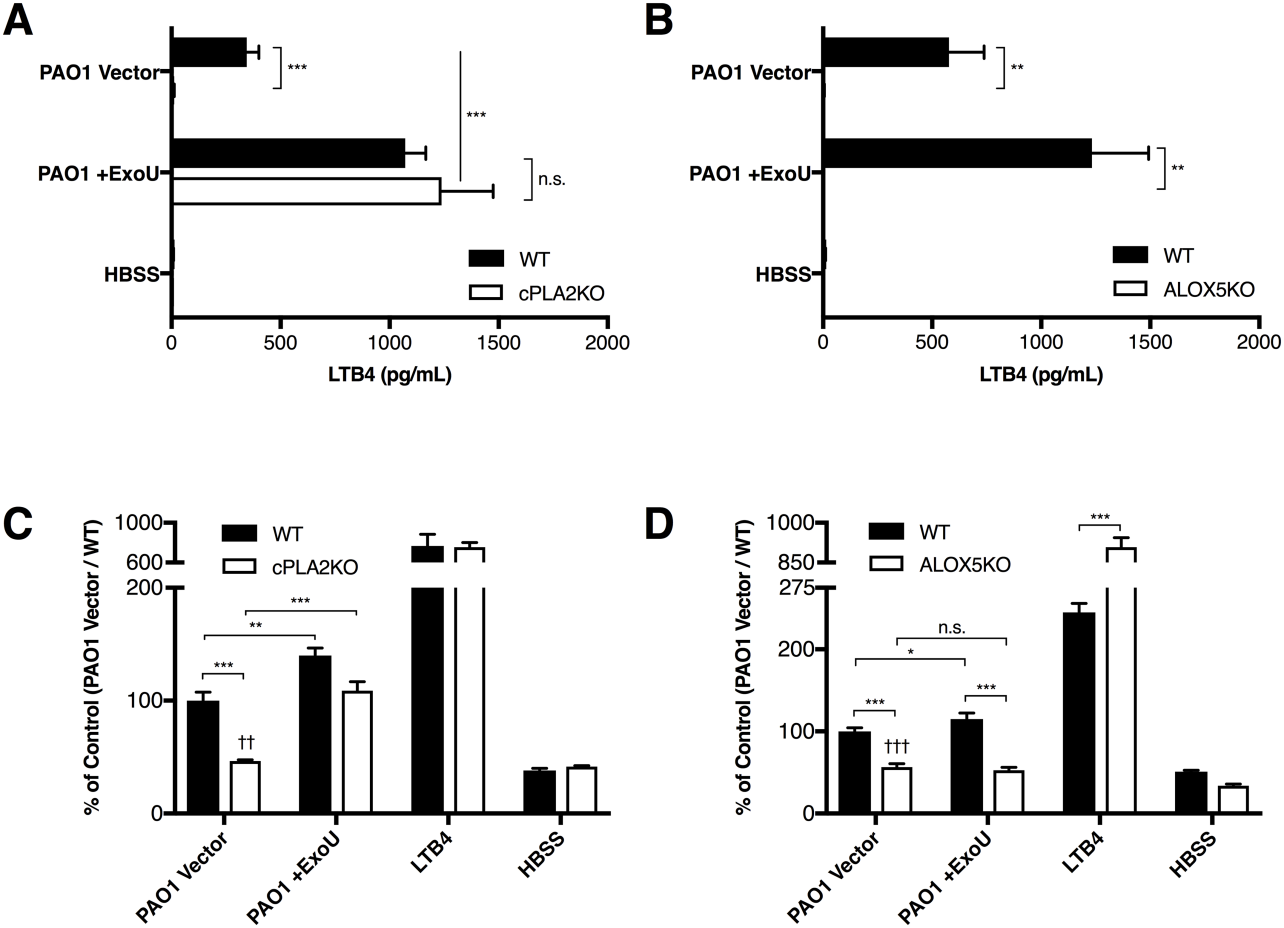
ExoU compensates for absence of cPLA2α, but not 5-LOX in a mouse in vitro model of neutrophil transepithelial migration. Mouse lung epithelial MLE12 monolayers were grown on inverted transwells and infected with paired PAO1 strains that express or lack ExoU. Bone marrow from (A, C) cPLA2α-/- mice or their littermate controls, or from (B, D) ALOX5-/- or WT mice were provided in the basolateral chamber to assess migration of bone marrow neutrophils. (A, B) Apical supernatant was assayed for total LTB_4_ content following 2h migration period. (C, D) Migration of neutrophils was assessed following epithelial infection with PAO1 Vector or PAO1 +ExoU. Neutrophil migration was reported as percent of control. The neutrophil migration response of WT neutrophils to the parental strain PAO1 Vector was set to 100%. Data are shown as mean +/- SD, and are representative of multiple independent experiments. *p ≤ 0.05, **p < 0.01, ***p < 0.001 between the indicated conditions. n.s. indicates a non-statistically significant difference. ††p <0.01, †††p <0.001 indicates comparison to HBSS control.

As in the human H292 co-culture model, we observed apical LTB_4_ release after wild type neutrophils migrated in response to epithelial infection (Fig 4A, black bars). A significant increase in LTB_4_ was associated with migration in response to epithelial infection with PAO1 +ExoU as compared to PAO1 Vector. When performing this experiment using bone marrow neutrophils of cpla2α-deficient littermates, only background levels of LTB_4_ were detected in response to neutrophil migration to epithelial infection with PAO1 vector. Neutrophils migrating in response to PAO1 +ExoU, on the other hand, effectively produced apical LTB_4_ (Fig 4A, white bars). Migrating neutrophils from 5-LOX-deficient mice failed to produce apical LTB_4_ in response to any condition (Fig 4B).

Total migration correlated strongly with apical LTB_4_ generation. cPLA2-deficient neutrophils migrated poorly in response to epithelial infection with PAO1 Vector, although migration was observed slightly above background levels (Fig 4C). In contrast, cPLA2-deficient neutrophils migrated significantly more effectively in response to epithelial infection with PAO1+ExoU as compared to PAO1 Vector. The restoration of migration with the cPLA2-deficient neutrophils was also observed in response to MLE12 infection with PA14 relative to infection with PA14ΔexoU (S7 Fig). ALOX5^-/-^ bone marrow neutrophils had similar deficiencies when migrating in response to epithelial infection. However, in contrast to cPLA2α^-/-^ bone marrow, the presence of ExoU had no significant impact in restoring neutrophil migration deficits (Fig 4D).

Taken together, these data indicate that ExoU expression in the pathogen is capable of complementing cPLA2α deficiencies in the neutrophil. This resulted in significant restoration of LTB_4_ generation, as well as subsequent restoration of neutrophil migration. ExoU was unable to complement deficiencies in ALOX5, suggesting that ExoU functions as a PLA2, and not as a general stimulant of LTB_4_ production.

### ExoU expression is associated with increased LTB_4_ and PMN infiltration independent of ExoU-mediated cytotoxicity in vivo, modeling the early stages of acute pneumonia

**Fig 5:**
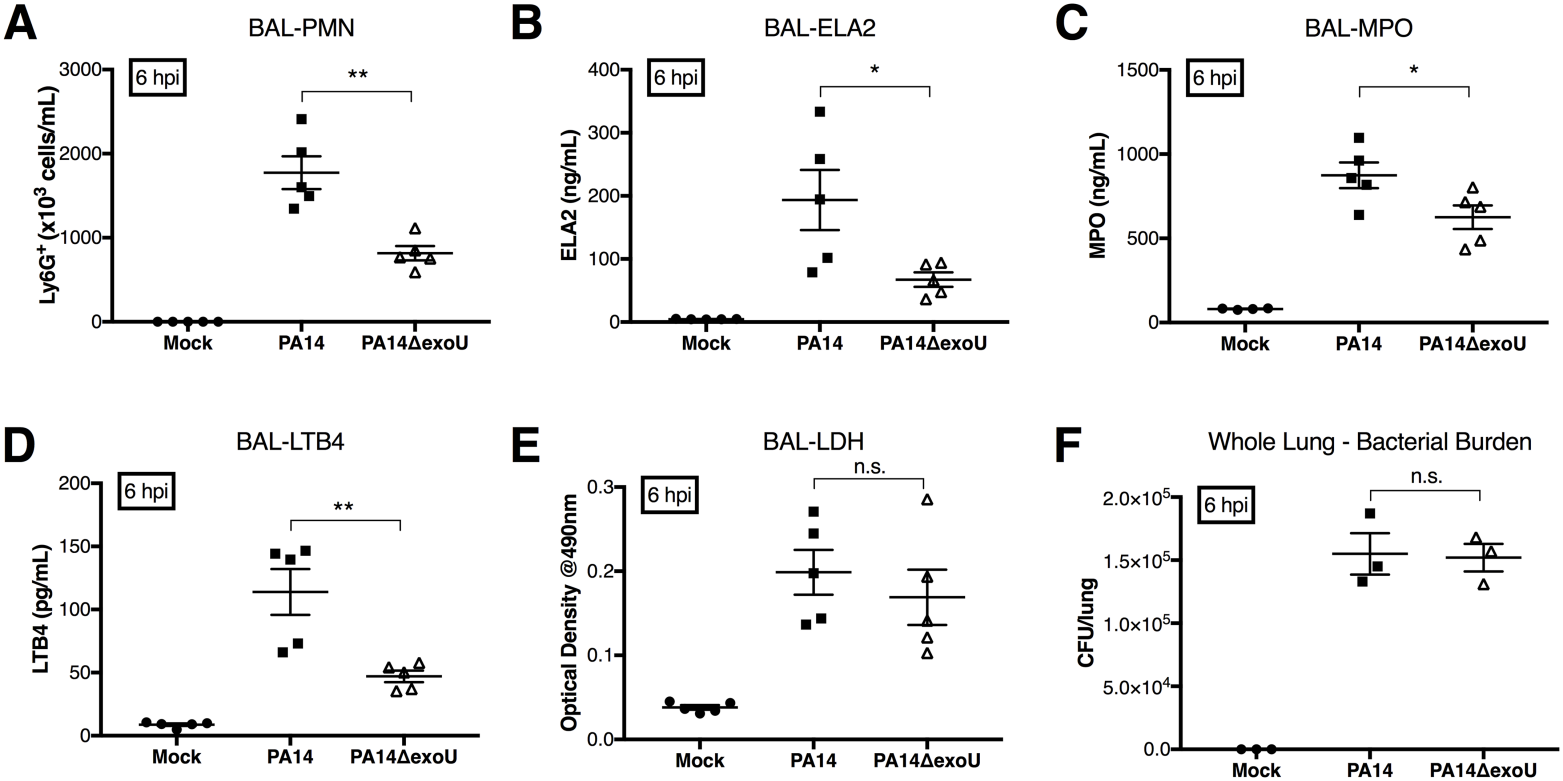
ExoU expression is associated with increased LTB_4_ and PMN infiltration independently of any detectible ExoU-associated cytotoxicity at early stages in an in vivo model of acute pneumonia. Adult 6–8 week old female C57BL/6J mice were challenged by intranasal inoculation of either PA14 or PA14ΔexoU. Bronchial alveolar lavage (BAL) samples were collected at 6hpi and total cell counts were performed. (A) Total cells were stained for Ly6G and analyzed by flow cytometry. Protein extracts were prepared from BAL samples and analyzed for (B) neutrophil elastase/ELA2 and (C) myeloperoxidase/MPO. (D) BAL samples were spun down and supernatant liquids were tested for levels of LTB_4_ via ELISA. (E) LDH levels were assessed in the cell-free supernatant to assess cytotoxicity. (F) In independent experiments, 6–8 week old C57BL/6J female mice were infected and lung tissues were harvested 6hpi post infection to assess bacterial burden. Data are representative of five animals per condition and are shown as mean +/- SEM, and are representative of multiple independent experiments. *p ≤ 0.05, **p < 0.01, indicate comparison between PA14 and PA14ΔexoU. n.s. indicates a non-statistically significant difference.

Having demonstrated that ExoU is capable of modulating neutrophil recruitment in an *in vitro* co-culture model, we sought to evaluate the mechanism in a dynamic *in vivo* context. Mice were acutely infected with either PA14 or PA14ΔexoU, a strain with a deletion of the ExoU gene. At 6hpi, bronchoalveolar lavage was performed for analysis of neutrophil infiltration to the lumen. PA14 infection was associated with increased neutrophil infiltration, as measured by flow cytometry when compared to PA14ΔexoU (Fig 5A). We additionally detected increased levels of neutrophil elastase (Fig 5B) and myeloperoxidase (Fig 5C) in the airways of mice infected with PA14. We also observed a significant increase in LTB_4_ release in lavage fluid (Fig 5D). At this 6hpi time point, we did not observe significant differences in cytotoxicity as measured by release of LDH (Fig 5E), or bacterial burden (Fig 5F).

ExoU was previously shown to enhance cytotoxicity as well as bacterial burden in the lungs of mice infected with some strains of *P*. *aeruginosa* [39]. At a later stage of the acute pneumonia process, 18hpi, we did observe more pronounced cytotoxicity and a greater bacterial burden in mice infected with the PA14 strain, in line with previous observations (S8 Fig). Taken together, these data support the hypothesis that ExoU mediates enhanced release of LTB_4_ resulting in exacerbated neutrophil infiltration of the lungs independently of its cytotoxotic functions.

## Discussion

*P*. *aeruginosa* is a ubiquitous gram negative pathogen that typically causes disease in immune compromised patients. Chronic *P*. *aeruginosa* infection of the airway is a significant source of morbidity and mortality in patients with CF. Infection results in recurrent waves of neutrophilic inflammation, causing progressive damage to the airway. In addition to CF, neutrophil-mediated pathology of the airway is a key contributor to morbidity associated with acute bacterial pneumonia, acute respiratory distress syndrome (ARDS), chronic obstructive pulmonary disease (COPD), severe asthma, and others. Controlling neutrophilic breach of the airway epithelium is a critical immunological check point of immense therapeutic value.

ExoU has been recognized as a significant virulence factor in *P*. *aeruginosa* infection and is often associated with worse clinical outcomes, severe disease, and increased neutrophil burden [29,40,41]. Once activated by ubiquitin transfer, ExoU is cytolytic in a wide variety of mammalian cell types, including epithelial cells and neutrophils [19,24,28,37]. ExoU also directly activates immune signaling pathways, increasing its virulence [32,42].

ExoU-associated cytolysis is mediated by lipase functionality [26,43] featuring both phospholipase A2 and lysophospholipase activity [27,44]. The use of phospholipase activity as a cytolytic defense strategy is one employed both by bacteria [30], as well as mammals [45]. In addition to inducing cytolysis, ExoU PLA2 function has been associated with modulating AA availability and subsequent eicosanoid generation, in particular production of PGE_2_ [31,32]. Given the importance of eicosanoid generation in neutrophil recruitment signaling pathways, we investigated how infection with ExoU-expressing strains of *P*. *aeruginosa* may impact this important inflammatory axis.

Epithelial infection with *P*. *aeruginosa* initiates the basolateral-to-apical migration of neutrophils by inducing the sequential production of eicosanoid chemoattractants [12]. Upon infection, pulmonary epithelia apically directs the secretion of the neutrophil chemoattractant HXA_3_ [11]. HXA_3_ has also been implicated in initiating neutrophil transepithelial migration in models of gastrointestinal epithelia [10,46,47]. Although HXA_3_ does require PLA2 activity in general, the cPLA2α isoform is not required [16]. ExoU expression in infection strains did not appear to have an impact on epithelial-derived HXA_3_ production. This reinforces prior observations that ExoU has significant functional overlap with cPLA2α, and may suggest ExoU has selective PLA2 function and selective eicosanoid-generating capacity in mammalian cells. Notably, others have observed that ExoU can induce epithelial production of the neutrophil chemoattractant IL-8 [32], however IL-8 gradients do not significantly impact the most distal step of neutrophil recruitment to the airspace, transepithelial migration [48].

Neutrophil contribution to their own recruitment through the production of LTB_4_ has been observed in various models [12,49,50], but pathogenic appropriation of this process has not been described. Here we describe a mechanism by which ExoU contributes PLA2 functionality to host neutrophils, amplifying LTB_4_ release and subsequent neutrophil recruitment. In this co-culture model of transepithelial neutrophil migration, ExoU expression was associated with increased production of LTB_4_. LTB_4_ is uniquely produced by neutrophils in this *in vitro* model system [12], though similar mechanisms may also play a role in other cell types, such as macrophages, *in vivo*. In this model neutrophils were not directly infected, although it is likely they come in direct contact with epithelial-attached bacteria once initial neutrophils migrate in response to epithelial HXA_3_ signals. Preferential injection of ExoU into recruited neutrophils and other phagocytes has been observed [28].

Typically, ExoU injection into neutrophils is associated with cytolysis, both *in vitro* and *in vivo* [37]. We optimized our model systems to maximize the viability of our cells in the presence of ExoU, to avoid the confounding impact of epithelial disruption and neutrophil cytolysis. It is likely that this neutrophil recruitment mechanism may work in concert with cytolysis to support bacterial growth [3]. *P*. *aeruginosa* can repurpose neutrophil products such as actin and DNA to encourage biofilm development [33]. Biofilm formation enhances the ability of the pathogen to resist neutrophil mobility, enzyme production, and oxidative burst [51]. The appropriation of neutrophil infiltration for the benefit of the pathogen is thought to play a particularly important role in the etiology of CF. Neutrophil-derived inflammatory mediators provide a metabolic advantage to *P*. *aeruginosa* [34], and *P*. *aeruginosa* manipulates eicosanoid signaling mechanisms to inhibit host resolution machinery [35]. This may explain why ExoU expression is associated with acute pulmonary exacerbations, but wanes in chronic infection [3,52]. Amplification of neutrophil recruitment to the airways, followed by cytolysis of the recruited innate immune cells may represent a pathogenic strategy that may benefit the development of bacterial persistence and provide a competitive advantage over other microbes in susceptible hosts.

cPLA2α is the rate-limiting step in eicosanoid biosynthesis, regulated at both the transcriptional and post-translational levels, and represents a key inflammatory checkpoint [15]. The eicosanoid storm, a pathological, inflammasome-mediated cascade of pro-inflammatory lipids, is the result of excessive activation of cPLA2α [53]. cPLA2α has been suggested as a potential therapeutic target to moderate pathophysiology of pulmonary inflammation [54], and endogenous cPLA2α function is associated with increased mortality in a murine model of acute *P*. *aeruginosa* pneumonia [55]. We show that ExoU is capable of circumventing endogenous cPLA2α regulation, and overcoming pharmacological inhibition or genetic ablation of cPLA2α function. Notably, therapies associated with modulating cPLA2α synthesis, such as glucocorticoids [56], may not be optimally effective at limiting ExoU-associated PLA2 function. Direct pharmacological targeting of cPLA2α function also impairs ExoU function [23,44,57] and may represent a valuable strategy. We isolated cPLA2α function from ExoU activity by targeting phosphorylation-mediated activation by the MAP/ERK pathway in our *in vitro* model. ExoU expression correlated with the recovery of LTB_4_ production by MAP/ERK-inhibited neutrophils. Similarly, ExoU expression was associated with a significant rescue of LTB_4_ production in mouse neutrophils genetically deficient for cPLA2α.

The ability of ExoU-expressing *P*. *aeruginosa* to overcome suppression or absence of cPLA2α suggests that the PLA2 activity associated with this cytotoxin is a key component of ExoU-mediated inflammation and pathology. ExoU-enhanced neutrophil production of LTB_4_ results in increased transepithelial migration, and likely greater accumulation of neutrophils within the airspace. This represents a new pathological mechanism ascribed to the well-studied exotoxin that is likely to contribute to our understanding of its role in disease processes. Furthermore, as anti-inflammatory targets are identified, it will be important to understand host-pathogen interactions that may mitigate potential benefits of anti-inflammatory therapies. These findings underscore the importance of understanding mechanisms of neutrophilic breach at infected mucosal surfaces.

**Fig 6:**
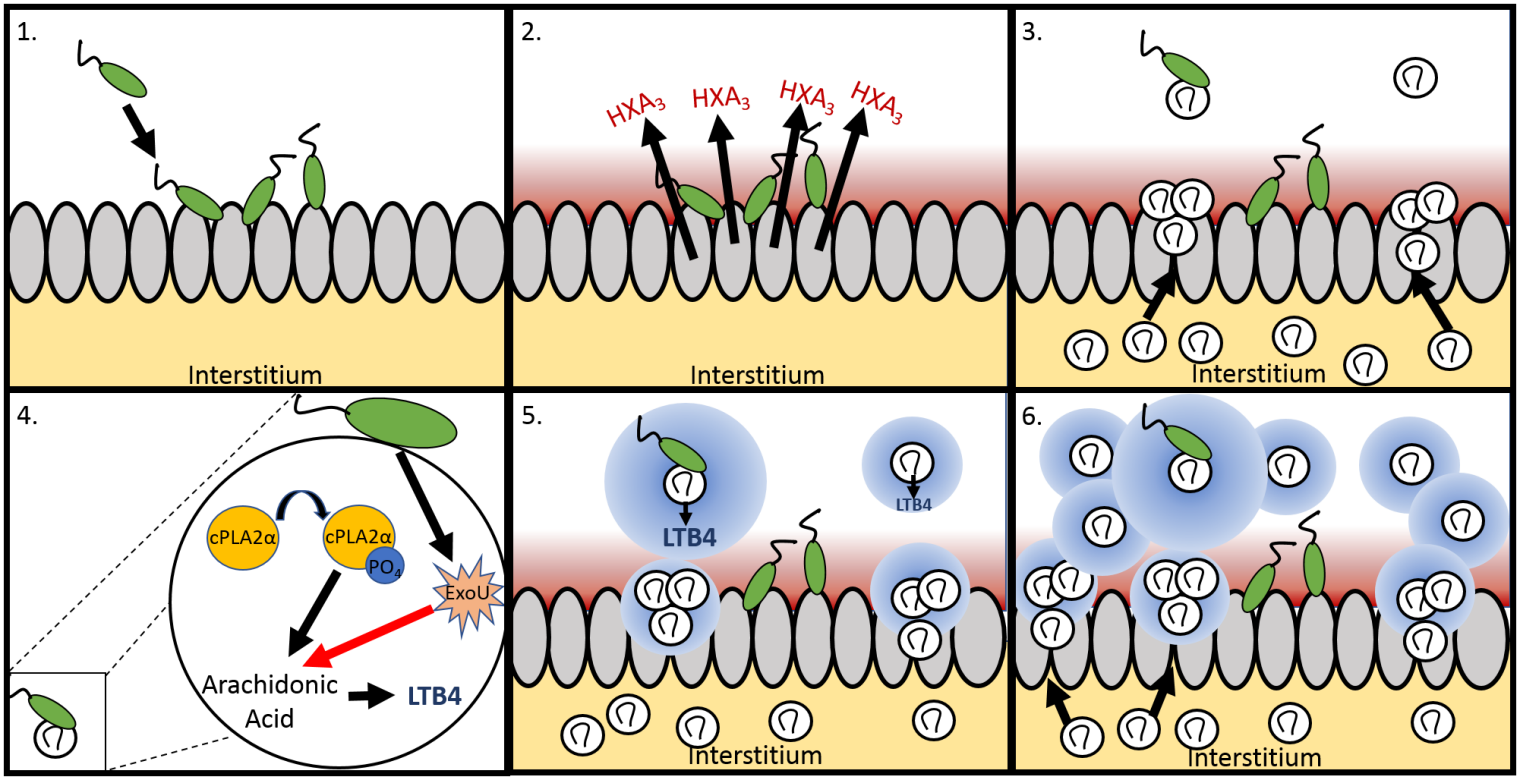
Schematic of the ExoU mechanism of enhancing LTB_4_ synthesis during neutrophil trans-epithelial migration. This schematic depicts (1) bacterial infection of lung epithelial cells and the resulting (2) HXA_3_ gradient which is produced apically from infected epithelial cells and independently of ExoU. (3) A small population of neutrophils respond to the HXA_3_ gradient and migrate across the epithelium into the airspace. Some of these neutrophils contact infecting *P*. *aeruginosa* cells (4), which in turn results in the injection of ExoU into the transmigrated neutrophil. Migrated neutrophil-derived cPLA2α liberates arachidonic acid from lipid membranes to be acted upon by 5-lipoxygenase for the generation of LTB_4_ and the introduction of bacterial ExoU into the neutrophil augments this process by generating additional arachidonic acid thereby amplifying the generation of LTB_4_ (5). This release of LTB4 by post migrated neutrophils and augmented by ExoU bolsters the chemotactic gradient driving more neutrophils across the epithelial barrier into the airspace enhancing the magnitude of the inflammatory response.

In summary, we present evidence to suggest that ExoU delivery into both epithelial cells and neutrophils by *P*. *aeruginosa* can directly modulate selective host eicosanoid generation through its PLA2 activity. In the context of neutrophil recruitment, the functional consequence of ExoU injection into neutrophils is the amplification of LTB4 release by neutrophils migrating across the epithelial barrier. By augmenting host cPLA2α activity, ExoU delivered by an infecting strain of *P*. *aeruginosa* bolsters a key chemotactic signal resulting in an intensified inflammatory response (Fig 6). Future studies exploring the *in vivo* acute pneumonia model and leveraging pharmacological intervention strategies and/or mouse gene deletion approaches have tremendous potential for more definitively resolving the contribution of ExoU during the initial stages of disease.

## Materials and methods

### Ethics statement

Whole blood was collected from healthy human volunteers after documenting written informed consent by signature on an approved consent form as part of a study protocol approved following review by the Institutional Review Board of Massachusetts General Hospital (protocol number 1999P007782, human subjects assurance number 00003136). Animal experiments were performed on female mice aged 6–8 weeks in strict accordance with the Guide for the Care and Use of Laboratory Animals of the National Institutes of Health. The protocol was reviewed and approved by the Institutional Animal Care and Use Committee of Massachusetts General Hospital (Protocol 2011N000104, animal welfare assurance number A3596-01). Animals were maintained under specific pathogen free conditions. Animals used in the acute pneumonia model were anesthetized by intraperitoneal injection with tribromoethanol, and euthanized under anesthesia by cervical dislocation. For bone marrow collection, bone marrow was harvested from mice following euthanasia by controlled-flow carbon dioxide asphyxia as per recommended guidelines.

### Bacterial strains

The PAO1 strain containing the pUCP19 vector plasmid (PAO1 Vector) and the PAO1 strain containing the vector plasmid expressing ExoU, its chaperone SpcU, and flanking DNA (PAO1 +ExoU) were kindly provided by Dr. Gregory Priebe. The generation of these strains was previously described [21]. The PA14ΔexoU strain was produced according to methods previously described [58]. Briefly, internal regions of ExoU were amplified by PCR from PA14 using 5’-GCGCCTGCAGGGCTCCGGAGTCACCTTT -3`and 5`-GCGCGGATCCCCCTCGAACTCAAGGATCAG -3`. Each primer contained restriction sites for ligation of PCR product into the integration vector pEXG2 using Promega T4 DNA ligase [59]. A PA14ΔExoU clean deletion mutant was obtained from Dr. Frederick Ausubel. All *P*. *aeruginosa* strains were grown aerobically in Luria—Bertani broth overnight (37°C, 225 RPM). PAO1 Vector and PAO1+ExoU cultures were grown in the presence of carbenecillin (400μg/mL). PA14ΔexoU-pEXG2 (PA14 –ExoU) cultures were grown in the presence of gentamycin (50μg/mL), KH_2_P0_4_ (5mM) and K_2_HP0_4_ (5mM). All strains were tested for the presence of ExoU via western blot to verify that deletion strains or strains that do not naturally possess this gene lacked the protein and to quantify the differential expression of ExoU between PA14 and PAO1+ExoU (S5 Fig). Prior to infection, 1 ml of overnight culture was pelleted (16,000 x g, 5 minutes), washed with 1 ml of Hank’s balanced salt solution with calcium and magnesium (HBSS+), re-pelleted (16,000 x g, 5 minutes), and re-suspended in 600μl of HBSS+. The optical density for each strain was obtained at 600nm using the Spectramax M5 (Molecular Devices Corp., Sunnyvale, Ca), and were set equal. The stock solutions of each strain were used to make the appropriate dilutions. Optical density was correlated to bacterial concentration dilution plating and development of a standard curve for each strain.

### Lung epithelial culture

Human A549, human H292, and murine MLE12 lung epithelial cells were purchased from the ATCC. Generation of stably transfected A549 cell lines housing either a control plasmid or *cpla2α* RNAi-expressing plasmid were previously described [16]. Cell lines were maintained in DMEM/F12 (1:1) culture media containing 10% heat-inactivated serum and 1X antibiotics. A549 cells were grown for 5-7d on 24w plates for AA and PGE_2_ release assays, and 162cm^2^ flasks for lipid bioactivity assays. Preparation of the transwell system was previously described [60]. Transwell inserts were flipped to an inverted position and collagen coated with a 30 μg/ml collagen solution. Collagen coating was dried for 4 h. The collagen-coated underside of the transwell was seeded with lung epithelial cells and allowed to attach overnight. Cultures were then placed in DMEM:F12 (1:1) with 10% heat-inactivated serum and 1x antibiotics and grown to confluence. MLE12 cultures were maintained for 3–5 days after seeding, and H292 cultures were maintained for greater than 7 days. Monolayer integrity was confirmed by maintenance of fluid resistance between apical and basolateral compartments.

### Detection of cPLA2α expression

A549 lung epithelial cells expressing anti-cPLA2α or irrelevant control RNAi were seeded on 6-well plates and grown to confluence. Cells were scraped off in buffer containing 150 mM Tris pH 8.0, 15 mM EDTA, 6 mM EGTA, 200 mM PMSF, 4 mM Na_3_VO_4_, 40 mM NaF, and 1 Complete Mini protease inhibitor cocktail tablet/10 ml buffer. Cells were then sonicated, followed by centrifugation at 55,000 rpm for 1 h. Pellets were then resuspended in lysis buffer (0.1% Triton-X-100, 0.2% SDS, 50 mM Tris pH 8.0, 5 mM EDTA, 2 mM EGTA, 200 mM PMSF, 4 mM Na3VO4, 40 mM NaF, and 1 Complete Mini protease inhibitor cocktail tablet/10 ml buffer). Lysates were again sonicated and centrifuged at 30,000 rpm for 10 min. Supernatants were collected and concentrated using Centricon filters (30 KDa cuttoff). Total protein concentration of lysate samples were normalized and electrophoresed on an 8–16% gradient polyacrylamide gel (Bio-Rad Laboratories, Hercules, CA) and transferred to nitrocellulose. Blots were probed with rabbit anti-GAPDH (Santa Cruz Biotechnology), or anti-cPLA2 antibody (Cell Signaling Technology). ECL reagent was used to detect protein following incubation with HRP-conjugated goat anti-rabbit antibody. Relative levels of protein detected were quantified by densitometry analysis.

### AA release assay

AA release was assayed as previously described [38,61]. A549 cells were washed three times, and treated with media containing 0.2μCi/ml of tritiated arachidonic acid ([^3^H]AA). Cells were incubated for 18–24h to allow incorporation of [^3^H]AA. After incubation, cells were washed three times and infected with 0.5ml of bacteria (6x10^7^CFU/ml). Supernatants were collected and measured by scintillation counting. At the completion of the assay, cells were solubilized in 1% SDS and 1% Triton X-100 and then sampled (250μl) for measurement of total radioactivity by scintillation counting. Data is presented as percent release of total cpms.

### Human neutrophil isolation

Whole blood was anti-coagulated with acid citrate/dextrose, and buffy coats were obtained by centrifugation at 400xg at room temperature. Plasma and mononuclear cells were removed by aspiration, and most RBCs were removed by 2% gelatin sedimentation. Residual RBCs were lysed using a cold ammonium chloride buffer. After lysis, neutrophils were washed and resuspended in HBSS without calcium or magnesium (HBSS-) at 5x10^7^/mL.

### Transmigration assay

Neutrophil transepithelial migration was assayed as previously described [60]. Lung epithelial monolayers were grown inverted on 24w or 96w transwell inserts (Corning Life Sciences). Transwells were first washed and equilibrated in HBSS+ for 30 min prior to infection. After equilibration, transwells were again inverted and the apical surface of the epithelial cells was infected with 25μL of bacteria. Unless otherwise indicated, *P*. *aeruginosa* infection concentration was 3-6x10^7^CFU/mL. After infection, transwells were washed and prepared for migration. *P*. *aeruginosa*-infected cells were placed in wells containing HBSS+, as were HBSS control wells. Chemotactic gradients of LTB_4_ (5 ng/ml; Enzo Life Sciences, Farmingdale, NY) and IL-8 (100ng/ml; eBioscience, San Diego, CA) were prepared in HBSS+ and provided at the apical chamber. Lipid fractions were prepared as described and serially diluted for optimal dosing. Neutrophils (2x10^5^/96w transwell, or 1x10^6^/24w transwell) or whole bone marrow cells (8x10^5^/96w transwell, 2x10^6^/24w transwell) were supplied to the basolateral chamber and incubated for 2h at 37°C. After a 2h migration, the transwells were removed and the apical well was assayed for neutrophil content by myeloperoxidase assay [60]. In experiments where different neutrophil treatments groups or bone marrow genotypes were used in the same assay, standard curves were used to control for any potential variation in preparation or myeloperoxidase activity. Migration values were normalized by setting the magnitude of the parental bacterial strain (i.e. PAO1 Vector or PA14)-induced neutrophil migratory response to 100%, within internally controlled experiments.

### Neutrophil inhibitor treatments

Neutrophil inhibitor treatments were performed immediately prior to infection. Neutrophils (2.5x10^7^/mL) were incubated with 20μM U0126 (Cell Signaling Technology), or 2μM MK886 (Enzo Life Sciences) for 1h at 37°C in HBSS-. After drug treatment, neutrophils were resuspended in HBSS+ containing inhibitors at 5x10^6^/mL, and promptly utilized in the transepithelial migration assay. Neutrophil viability following drug treatment was assessed by trypan blue exclusion. No evidence of decreased viability was observed compared to vehicle-treated or untreated neutrophil controls.

### Lipid extraction and bioactivity assay

Lipid extraction was performed as previously described [12,36]. Briefly, lung epithelial cells were seeded in a 162cm^2^ flask and grown to confluence. Confluent monolayers were washed and infected with the indicated strains at 6x10^7^CFU/mL or mock infected for 1h at 37°C. The monolayers were washed of non-adherent bacteria three times and then incubated for an additional 2h in HBSS+ at 37°C. Supernatants were collected and acidified to pH 5 and poured through a Supelco Discovery DSC-18 SPE column (Sigma-Aldrich) and eluted with methanol. This lipid fraction in methanol was dried under a stream of nitrogen and stored at -80°C for further processing. Immediately prior to the experiment, lipid fractions were resuspended in 1ml methanol, dried under a stream of nitrogen, then resuspended in HBSS+. Lipid fractions were then serially diluted and assessed by transmigration assay or LC/MS/MS.

### Eicosanoid detection

Eicosanoids were quantified by two distinct methods. Where noted, eicosanoid detection ELISAs for the quantification of LTB_4_ and PGE_2_ were purchased from Cayman Chemical (Ann Arbor, MI) and performed according to manufacturer’s instructions. Cellular supernatants were collected and stored at -20°C until assay was performed. LC/MS/MS was also performed on lipid-extracted supernatants by a triple quadrupole linear ion trap system (3200 QTRAP; AB SCIEX; Framingham, MA) as previously described [16,53].

### Viability and cytotoxicity assays

A549 epithelial cells were grown on a 24-well plastic dish to confluence. A549 cells were washed then infected with 0.5mL of the indicated bacterial strain and incubated for 1h at 37°C and 5% CO2. After 1h, the cells were washed three times and incubated in HBSS+ for an additional 3h. The positive control was incubated in 1% TX-100. Supernatant was then collected and analyzed by lactate dehydrogenase (LDH) release assay according to manufacturer’s instructions (Sigma). Cytotoxicity of neutrophils and mouse bone marrow were also assessed by LDH assay. 1.25x10^6^ neutrophils or 5x10^5^ bone marrow cells were suspended in 75μL, then infected with 25μL of bacteria at either ~3x10^9^ or ~3x10^7^ CFU/mL, or lysed with 1% Triton x-100 for 2h. After 2h, LDH release was assayed according to manufacturer’s instructions. To assess transwell-grown epithelial viability following infection, transwells were washed and infected with the indicated bacterial concentrations for 1h at 37°C. Cells were then washed, and incubated for 2h in HBSS+. Viability was then assessed by MTT Assay (Life Technologies) according to manufacturer’s instructions. MTT assays were selected to evaluate transwell-grown epithelial monolayers due to increased sensitivity when analyzing small volumes in 96-well transwells. LDH assays were selected to evaluate cytotoxicity of plate-grown epithelial cells, as well as neutrophils and mouse bone marrow in response to bacterial infection.

### Preparation of bacterial lysates

LB broth was inoculated with bacterial strains (PA14, PA14ΔexoU, PAO1 Vector or PAO1+ExoU) and incubated overnight at 37°C, 220RPM. 1mL of overnight culture was transferred into sterile 1.5mL Eppendorf tubes, and cultures were spun down for 5min at 13,000 RPM (Eppendorf Centrifuge 5424). Bacterial pellets were re-suspended and washed in 1mL of HBSS+. Cultures were spun again (5min, 13,000RPM) and the bacterial pellet was re-suspended in 1.4mL and optical density was obtained at 600nm. Bacterial O.D. was adjusted for all cultures to 0.8 and 1mL of O.D. adjusted culture was spun down (5min, 13,000RPM) and re-suspended in 250μL TE buffer containing 0.3mM PMSF. Bacterial cultures were sonicated (5W at 10s on, 15s off for 2 min total) on ice, and spun (16,000 x G, 5 min). Supernatant liquid was transferred to a new sterile 1.5mL Eppendorf tube and saved for further analysis via ExoU western blot. Protein levels were measured via a modified Lowry assay (Bio-Rad, CA).

### ExoU western blot

Protein lysates were set to have equal concentrations of protein/mL, and boiled for 5 min (100°C) with loading buffer. Samples were loaded into tris-glycine wedge acrylamide gels (Thermo Fisher, MA) and run at 100V for 2h. Proteins were transferred from the gel to 0.2μm nitrocellulose membranes via wet-transfer (25V, 3h). Membranes were blocked for 1h at 4°C in TBS buffer containing 5% BSA and 0.05% Tween 20. Membranes were washed 3X with TBS (5 min) and placed in primary antibody solution (10mL TBS, 10μL Tween-20, pinch of BSA and 1.5μL ExoU Ab U7.15 obtained from D.W. Frank, Ph.D. at the Medical College of Wisconsin). Membranes were incubated overnight at 4°C with constant agitation in primary Ab solution. After incubation, membranes were washed 3X (10 min) in TBS containing 1% Tween-20. Membranes were incubated for 1h at RT in anti-mouse HRP linked secondary Ab (25mL TBS, 25μL Tween-20, pinch of BSA, 10μL anti-mouse HRP Ab). Afterwards membranes were washed 3X again (TBS, 1% Tween-20) and then rinsed in TBS. Membrane was incubated in ECL- HRP RxN (Thermo Fisher, MA) solution for 1 min and then developed using the BioRad Universal Hood III.

### Mouse bone marrow collection

C57BL/6J wild-type and Alox5^-/-^ mice were purchased from The Jackson Laboratory (Bar Harbor, ME) and housed in specific pathogen-free conditions [62]. Mice were group housed from the point of arrival to the point of experimentation. Femurs and tibias from Balb/C cPLA2^-/-^ mice and wild-type littermate controls were obtained from Joseph Bonventure [63]. Femurs and tibia were collected and flushed with HBSS- to collect bone marrow. RBCs were lysed using a cold ammonium chloride solution. After lysis, remaining bone marrow was washed and counted immediately prior to use.

### Acute pneumonia model

C57BL/6J female mice were anesthetized by intraperitoneal administration of a freshly prepared mixture of tribromoethanol solution (25mg/ml, Thermofisher, Belgium). Tribromoethanol was dissolved in 2-methyl-2-butanol (1g/mL, Sigma, MA) and was subsequently diluted to working concentrations in nano-pure H_2_O. Mice were randomly assigned to experimental groups of mock infection, PA14 infection or PA14ΔexoU infection. With mice held at an upright position, intranasal inoculation of either sterile HBSS+, PA14 or PA14ΔexoU (45 μl, 1.3e^7^ CFU/mL) was administered by placing 15μl over the nostrils 3X. Subjects were placed in the recovery cage for either 6 or 18 hours. After incubation, mice were re-anesthetized.

### Processing of bronchoalveolar lavage samples

An 18G catheter (BD, MA) was used to gain entrance into the trachea, and the lungs were flushed four times with a total volume of 2mL of HBSS supplemented with 0.3% FBS and 300μM EDTA. Collected bronchial alveolar lavage (BAL) was transferred into a 15 mL conical falcon tube, and placed on ice for further processing. BAL samples were separated into three separate aliquots: 0.3, 0.5 and 0.7 mL. Cells within the 0.3mL aliquot were blocked with anti-CD16/CD32 (1:200; BD Biosciences, San Jose, CA) for 20 min on ice. Suspensions were then split into two 150μl samples, and were either stained with anti—Ly6G-allophycocyanin (eBioscience) or isogenic tag and washed after 20 min incubation at 4°C. Data were collected on a BD FACSCalibur cytometer (BD Biosciences) and analyzed with FlowJo software (Tree Star, Ashland, OR). For protein lysate aliquots, 0.5mL BAL aliquots were treated with 10μL protease inhibitor cocktail set III, EDTA-free (Calbiochem, MA) and 20μl of 10% Triton X-100 (Sigma, MA) and mixed by inversion for 20 minutes at 4°C. Samples were used for mouse neutrophil elastase/ELA2 and MPO ELISA (R&D Systems, MN) according to the manufacturers protocol. The 0.7mL BAL aliquots were centrifuged (400 RCF, 5 min, 4°C), and cell-free supernatant was collected and used assay LDH and LTB_4_ content as described.

### Bacterial recovery from mouse lungs

Anesthetized mice were sacrificed via cervical dislocation and whole lung was removed surgically without bronchoalveolar lavage. Tissue placed into 2 mL Eppendorf tubes which contained a 5mm metal bead with 1 mL of HBSS+ and stored on ice. Tubes were placed in the TissueLyser LT (Qiagen, Germany) (50Hz, 6.5 min for lung, 1.5 min for spleen). Lung homogenate were plated on *Pseudomonas* isolation agar plates (PIA) using the drop plate method. Plates were dried on the bench and placed at 37°C overnight. Colonies were counted, and the total CFU/mL recovered from the homogenized tissue samples was calculated.

### Quantification and statistical analysis

Results are expressed as mean +/- SD unless otherwise indicated. Data are representative of at least three independent datum points per condition with multiple experiments yielding similar results. Data from in vivo experiments are representative of at least four individual mice per condition with multiple experiments yielding similar results. Single comparisons were evaluated using an unpaired two-tailed Student’s t-test. Where noted, data were analyzed by a two-way ANOVA with a Bonferroni posttest. A p value ≤ 0.05 was considered significant.

## Supporting information

S1 Fig ExoU expression has limited impact on cytotoxicity at the selected dose within 3h of infection. A549 cells were grown to confluence on a 24-well dish. Epithelial cells were then infected for 1h with the indicated bacterial strain, mock infected with HBSS+, or lysed with 10% triton x-100. Bacterial infection was performed at the indicated concentration. Cytotoxicity of A549 cells was determined by lactate dehydrogenase (LDH) release. Data are shown as mean +/- SD, and are representative of multiple experiments. ***p = 0.0013 vs HBSS controls.
(TIFF)

S2 Fig RNAi inhibition of host cPLA2α results in a significant decrease in cPLA2α protein expression. Lysates were collected from A549 epithelial cells transfected with RNAi plasmid targeting cpla2α or control. (A) Lysates were electrophoresed and transferred to a nitrocellulose gel and probed for cPLA2α or GAPDH as control. (B) Relative protein levels were determined by densitometry and normalized to GAPDH levels. Data are representative of 3 independent lysate samples, and are presented as mean +/- SD. **p < 0.01.
(TIFF)

S3 Fig ExoU-associated PLA2 activity does not enhance production of epithelial HXA3. (A, B) Total HXA_3_ levels were measured in lipid-extracted supernatants by LC/MS/MS following infection of non-transfected H292 epithelial cells. Where indicated, n.s. indicates a non-statistically significant difference. (C, D) Supernatant was collected from infected non-transfected A549 cells and lipid components were extracted. The relative chemotactic bioactivity of this lipid fraction was assessed using a neutrophil transwell migration assay. The magnitude of neutrophil migration reflecting the amount of chemotactic bioactivity was reported as percent of control. The neutrophil migration response to undiluted lipids derived from epithelium infected with the parental strains PAO1 Vector or PA14 was set to 100%. Lipid extracts were serially diluted to assess the neutrophilic chemotactic response to the lipids and neutrophil migration was analyzed by two-way ANOVA. Data are represented as means +/- SD and are representative of multiple independent experiments. There were no statistically significant differences observed between the chemotactic bioactivity measured between lipids extracted from A549 cells infected with either ExoU+ or ExoU- strains at any dilution concentration of extracted lipids, however, bioactivity derived from all strains at all dilutions was significantly great than bioactivity derived from lipids extracted from mock-infected A549 cells.
(TIFF)

S4 Fig ExoU western blots of bacterial lysates prepared from PAO1 and PA14 strain sets. Bacterial lysates prepared from PAO1 vector, PAO1+ExoU, PA14ΔexoU and PA14 containing equal concentrations of protein (A) were probed for the presence of ExoU to verify that knockout strains lacked the presence of ExoU. (B & C) ExoU expression levels in PAO1+ExoU and PA14 were assessed via western blots and analyzed with ImageJ software. The results demonstrate that PAO1+ExoU produces ExoU at a 12.5-fold higher concentration than PA14. Data are represented as means +/- SD, ***p < 0.001.
(TIFF)

S5 Fig ExoU-expressing strains of *P*. *aeruginosa* do not induce significant cytotoxicity in human H292 epithelial cells grown on transwell supports or human primary PMN within 3h of infection. H292 lung epithelial monolayers were grown inverted on transwell supports then infected with paired PAO1 (A) and PA14 (B) strains that express or lack ExoU at the indicated concentrations (CFU/mL). Cellular viability was assessed by MTT assay, and compared to negative (HBSS) and positive controls (1% Triton). (C) Human primary neutrophils (1.25x10^6^/well) were suspended in bacteria cultures in HBSS+ for 2h at 37°C. Cellular cytotoxicity was then assessed by LDH release as a proportion of release following treatment with 1% triton. Data are shown as mean +/- SD, and are representative of multiple experiments.
(TIFF)

S6 Fig ExoU expression was not associated with a significant increase in cytotoxicity in mouse epithelial cells grown on transwell supports or mouse primary bone marrow during a 3h infection. MLE12 lung epithelial monolayers were grown on inverted transwell supports, and infected with (A) paired PAO1 and (B) PA14 strains that express or lack ExoU at the indicated concentration (CFU/mL). Cellular viability was assessed by MTT assay following 1h infection, wash, and a further 2h incubation. Triton-x 100 (1%) was used as a positive control, and mock infection with HBSS was used as a negative control. (C) Whole bone marrow cells (5x10^7^/well) were suspended in bacteria cultures at the indicated concentrations in HBSS+ for 2h at 37°C. Cellular cytotoxicity was assessed by LDH release, and compared to lysis with 1% triton x-100, or HBSS alone. Data are shown as mean +/- SD, and are representative of multiple independent experiments.
(TIFF)

S7 Fig ExoU under natural expression compensates for absence of cPLA2α in a mouse in vitro model of neutrophil transepithelial migration. Mouse lung epithelial MLE12 monolayers were grown on inverted transwells and infected with paired PA14 strains that express or lack ExoU. Bone marrow from cPLA2α-/- mice or their littermate controls were provided in the basolateral chamber to assess migration of bone marrow neutrophils. Neutrophil migration was reported as percent of control. The neutrophil migration response of WT neutrophils to the parental strain PA14 was set to 100%. Data are shown as mean +/- SD, and are representative of multiple independent experiments. **p < 0.01, ***p < 0.001 between the indicated conditions. n.s. indicates a non-statistically significant difference. † p <0.05, indicates comparison to HBSS control.
(TIFF)

S8 Fig ExoU expression is associated with enhanced cytotoxicity, bacterial burden, and increased neutrophilic inflammation in later stages in an in vivo model of acute pneumonia. Adult 6–8 week old female C57BL/6J mice were challenged by intranasal inoculation of either PA14 or PA14ΔexoU. Bronchial alveolar lavage (BAL) samples were collected at 18h and total cell counts were performed. (A) Total cells were stained for Ly6G and analyzed by flow cytometry. Protein extracts were prepared from BAL samples and analyzed for (B) neutrophil elastase/ELA2 and (C) myeloperoxidase/MPO. (D) BAL cells were pelleted and supernatant liquids were tested for levels of LTB_4_ via ELISA. (E) LDH levels were assessed in the cell-free supernatant to assess cytotoxicity. (F) In independent experiments, 6–8 week old C57BL/6J female mice were infected and lung tissues were harvested 18hpi post infection to assess bacterial burden. Data are representative of at least four animals per group and are shown as mean +/- SEM, and are representative of multiple independent experiments. *p < 0.05.
(TIFF)

S1 Table Raw data file. Lists raw data from all primary and supplemental figures.
(XLSX)
