# Supplementary figures and images for: Pseudomonas aeruginosa ExoU augments neutrophil transepithelial migration
Source: PLoS Pathog. 2017 Aug 3;13(8):e1006548. doi: 10.1371/journal.ppat.1006548 (PMC5557605; doi:10.1371/journal.ppat.1006548)

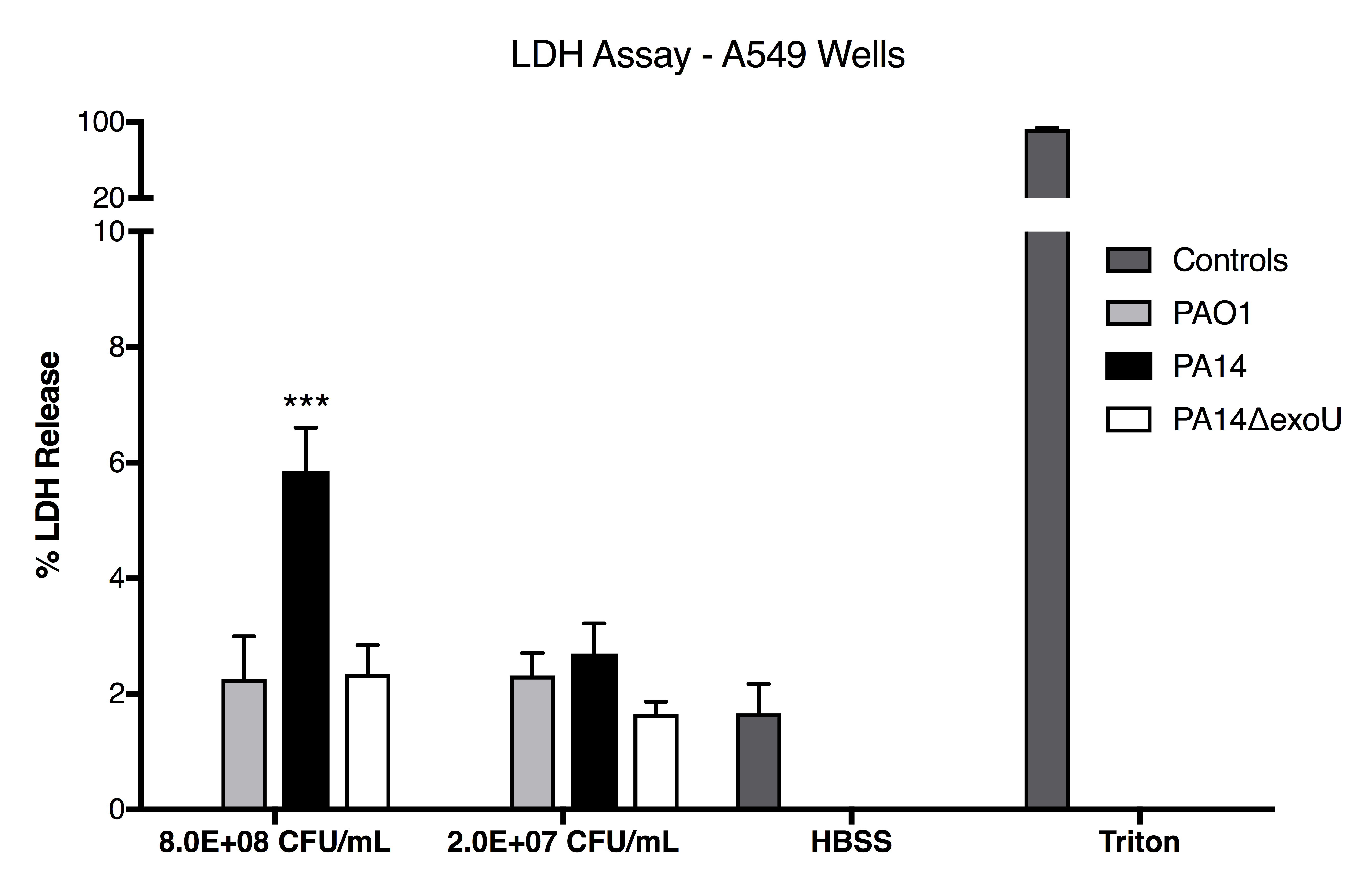

Supplement: S1 Fig — A549 cells were grown to confluence on a 24-well dish. Epithelial cells were then infected for 1h with the indicated bacterial strain, mock infected with HBSS+, or lysed with 10% triton x-100. Bacterial infection was performed at the indicated concentration. Cytotoxicity of A549 cells was determined by lactate dehydrogenase (LDH) release. Data are shown as mean +/- SD, and are representative of multiple experiments. ***p = 0.0013 vs HBSS controls. (TIFF) [file ppat.1006548.s001.tiff]

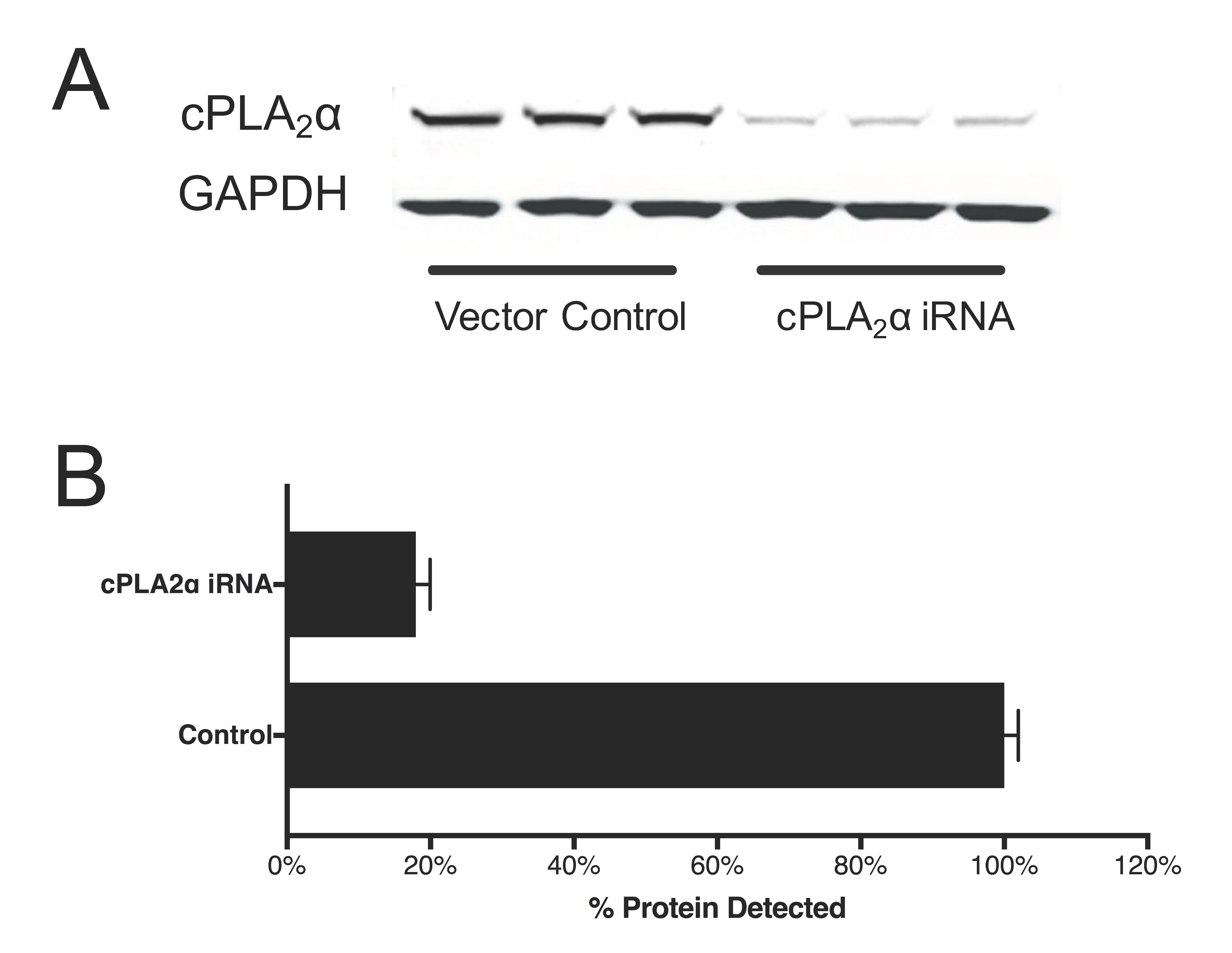

Supplement: S2 Fig — Lysates were collected from A549 epithelial cells transfected with RNAi plasmid targeting cpla2α or control. (A) Lysates were electrophoresed and transferred to a nitrocellulose gel and probed for cPLA2α or GAPDH as control. (B) Relative protein levels were determined by densitometry and normalized to GAPDH levels. Data are representative of 3 independent lysate samples, and are presented as mean +/- SD. **p < 0.01. (TIFF) [file ppat.1006548.s002.tiff]

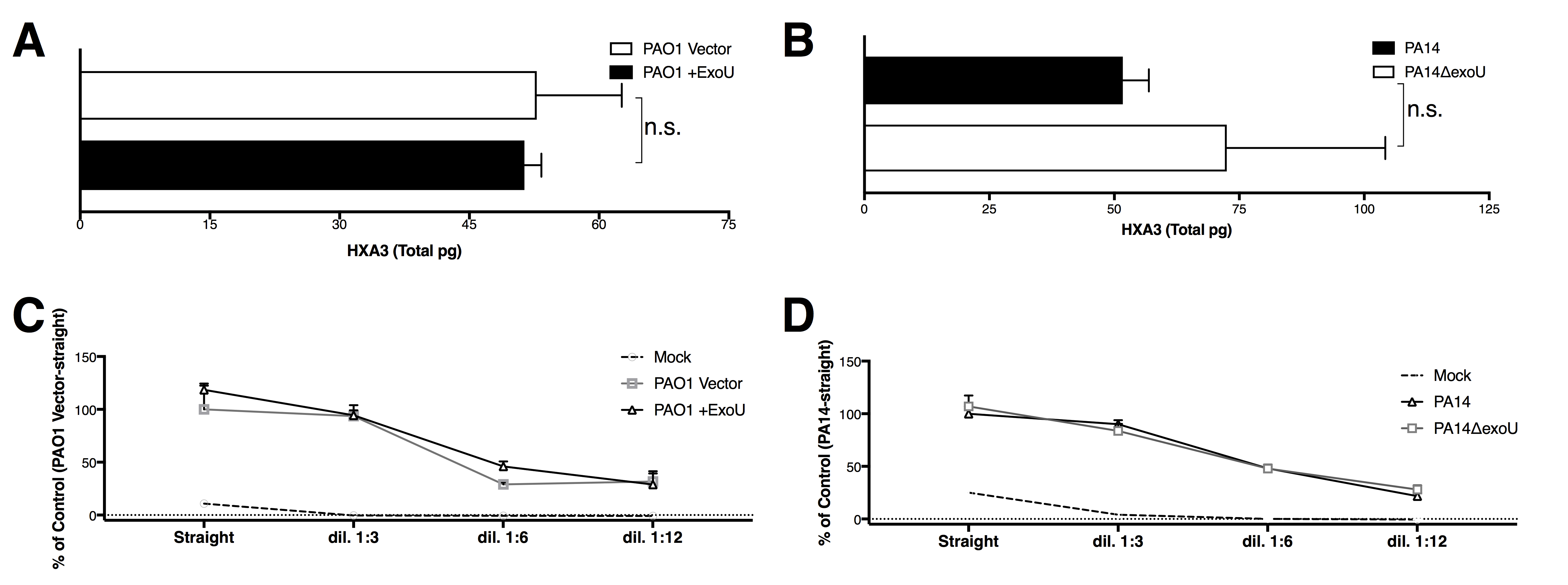

Supplement: S3 Fig — (A, B) Total HXA3 levels were measured in lipid-extracted supernatants by LC/MS/MS following infection of non-transfected H292 epithelial cells. Where indicated, n.s. indicates a non-statistically significant difference. (C, D) Supernatant was collected from infected non-transfected A549 cells and lipid components were extracted. The relative chemotactic bioactivity of this lipid fraction was assessed using a neutrophil transwell migration assay. The magnitude of neutrophil migration reflecting the amount of chemotactic bioactivity was reported as percent of control. The neutrophil migration response to undiluted lipids derived from epithelium infected with the parental strains PAO1 Vector or PA14 was set to 100%. Lipid extracts were serially diluted to assess the neutrophilic chemotactic response to the lipids and neutrophil migration was analyzed by two-way ANOVA. Data are represented as means +/- SD and are representative of multiple independent experiments. There were no statistically significant differences observed between the chemotactic bioactivity measured between lipids extracted from A549 cells infected with either ExoU+ or ExoU- strains at any dilution concentration of extracted lipids, however, bioactivity derived from all strains at all dilutions was significantly great than bioactivity derived from lipids extracted from mock-infected A549 cells. (TIFF) [file ppat.1006548.s003.tiff]

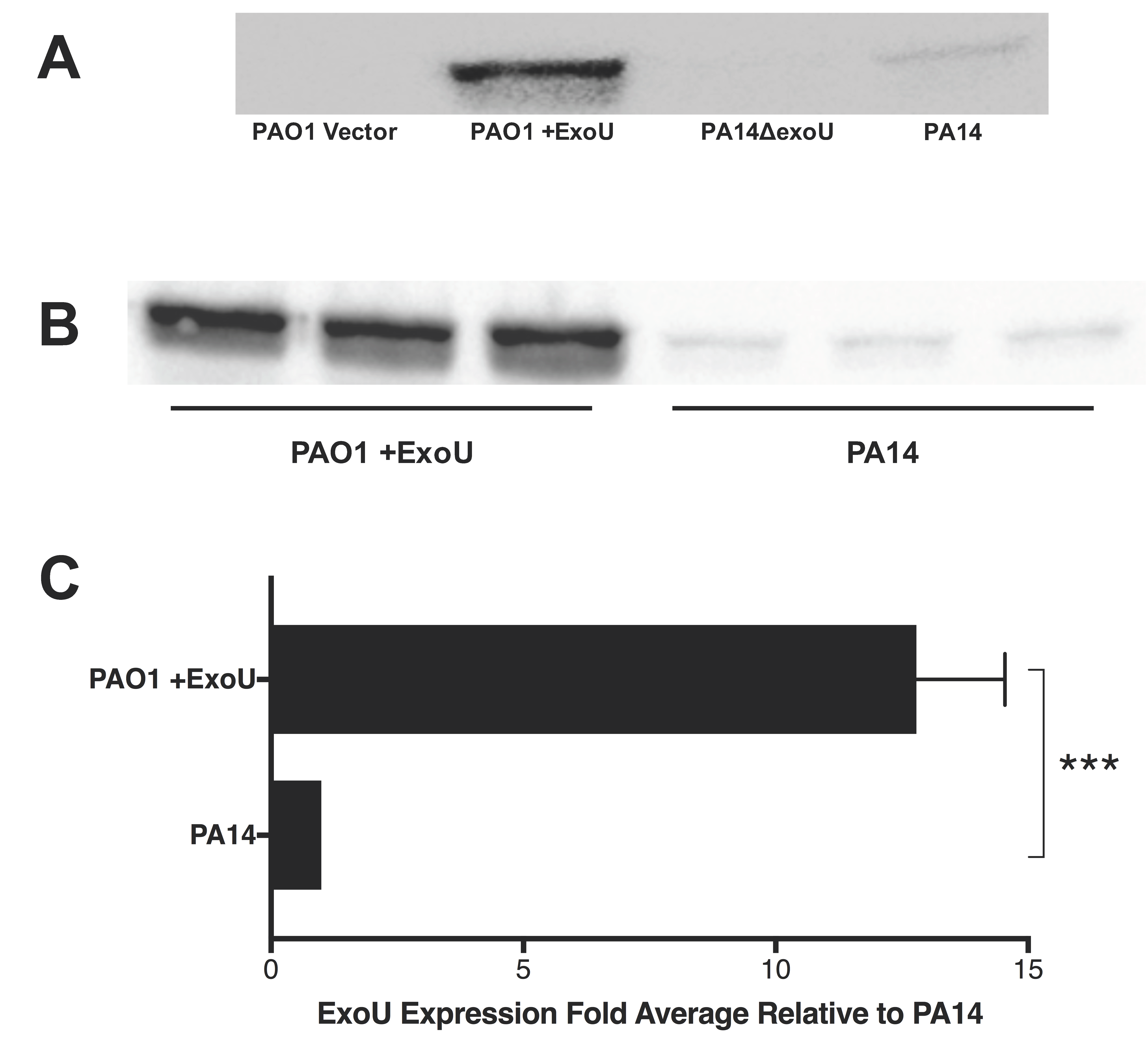

Supplement: S4 Fig — Bacterial lysates prepared from PAO1 vector, PAO1+ExoU, PA14ΔexoU and PA14 containing equal concentrations of protein (A) were probed for the presence of ExoU to verify that knockout strains lacked the presence of ExoU. (B & C) ExoU expression levels in PAO1+ExoU and PA14 were assessed via western blots and analyzed with ImageJ software. The results demonstrate that PAO1+ExoU produces ExoU at a 12.5-fold higher concentration than PA14. Data are represented as means +/- SD, ***p < 0.001. (TIFF) [file ppat.1006548.s004.tiff]

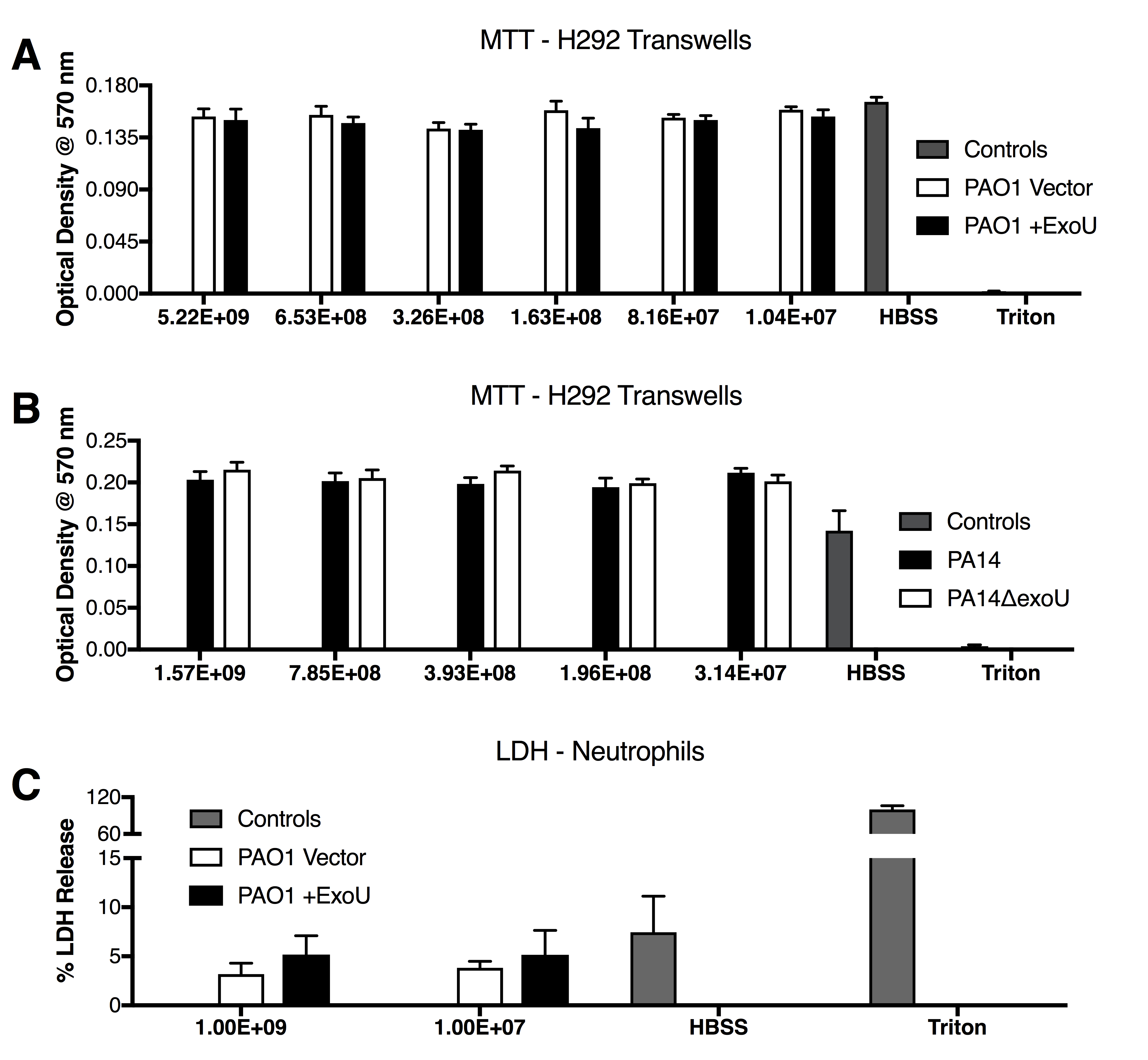

Supplement: S5 Fig — H292 lung epithelial monolayers were grown inverted on transwell supports then infected with paired PAO1 (A) and PA14 (B) strains that express or lack ExoU at the indicated concentrations (CFU/mL). Cellular viability was assessed by MTT assay, and compared to negative (HBSS) and positive controls (1% Triton). (C) Human primary neutrophils (1.25x10^6^/well) were suspended in bacteria cultures in HBSS+ for 2h at 37°C. Cellular cytotoxicity was then assessed by LDH release as a proportion of release following treatment with 1% triton. Data are shown as mean +/- SD, and are representative of multiple experiments. (TIFF) [file ppat.1006548.s005.tiff]

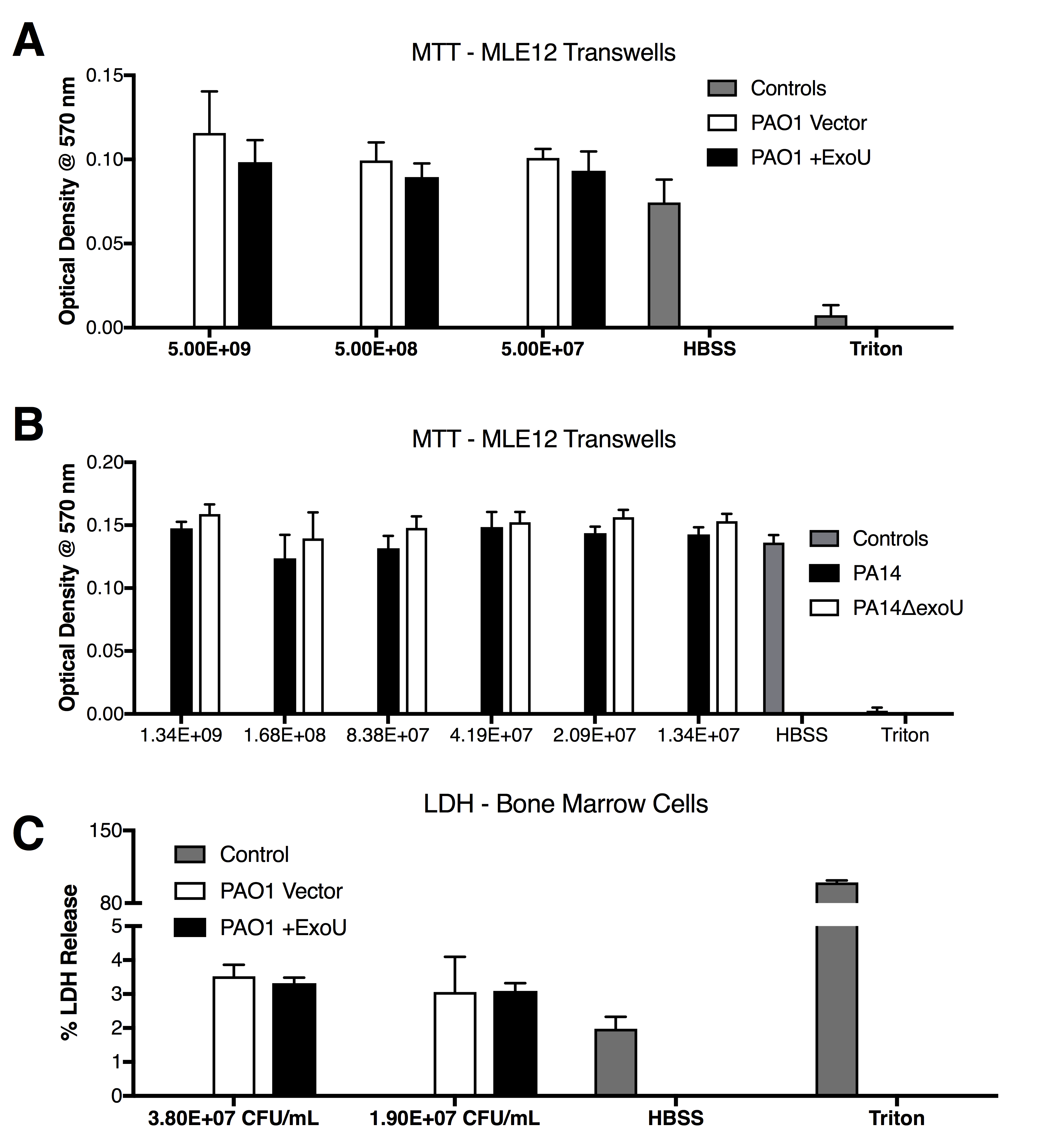

Supplement: S6 Fig — MLE12 lung epithelial monolayers were grown on inverted transwell supports, and infected with (A) paired PAO1 and (B) PA14 strains that express or lack ExoU at the indicated concentration (CFU/mL). Cellular viability was assessed by MTT assay following 1h infection, wash, and a further 2h incubation. Triton-x 100 (1%) was used as a positive control, and mock infection with HBSS was used as a negative control. (C) Whole bone marrow cells (5x10^7^/well) were suspended in bacteria cultures at the indicated concentrations in HBSS+ for 2h at 37°C. Cellular cytotoxicity was assessed by LDH release, and compared to lysis with 1% triton x-100, or HBSS alone. Data are shown as mean +/- SD, and are representative of multiple independent experiments. (TIFF) [file ppat.1006548.s006.tiff]

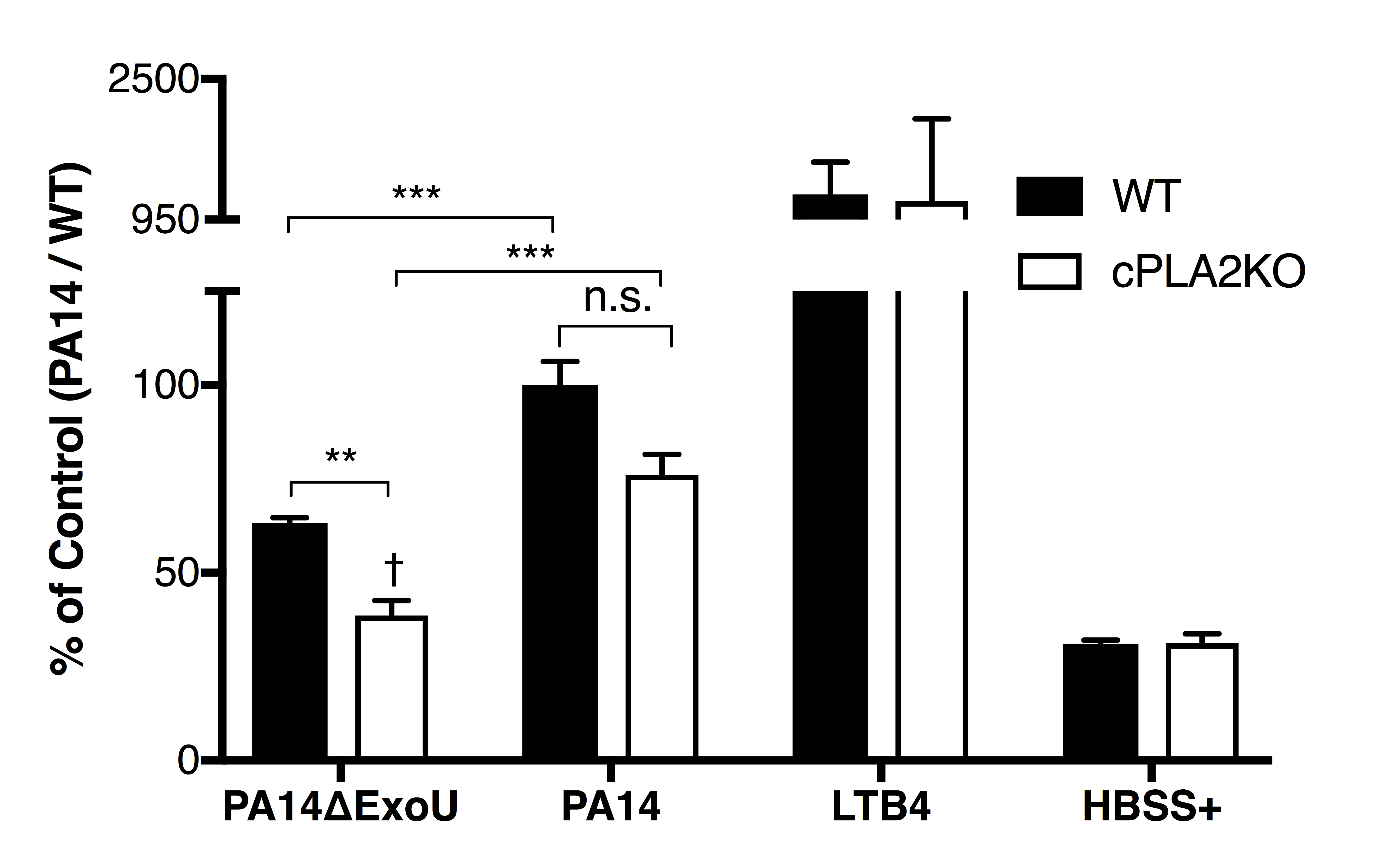

Supplement: S7 Fig — Mouse lung epithelial MLE12 monolayers were grown on inverted transwells and infected with paired PA14 strains that express or lack ExoU. Bone marrow from cPLA2α-/- mice or their littermate controls were provided in the basolateral chamber to assess migration of bone marrow neutrophils. Neutrophil migration was reported as percent of control. The neutrophil migration response of WT neutrophils to the parental strain PA14 was set to 100%. Data are shown as mean +/- SD, and are representative of multiple independent experiments. **p < 0.01, ***p < 0.001 between the indicated conditions. n.s. indicates a non-statistically significant difference. † p <0.05, indicates comparison to HBSS control. (TIFF) [file ppat.1006548.s007.tiff]

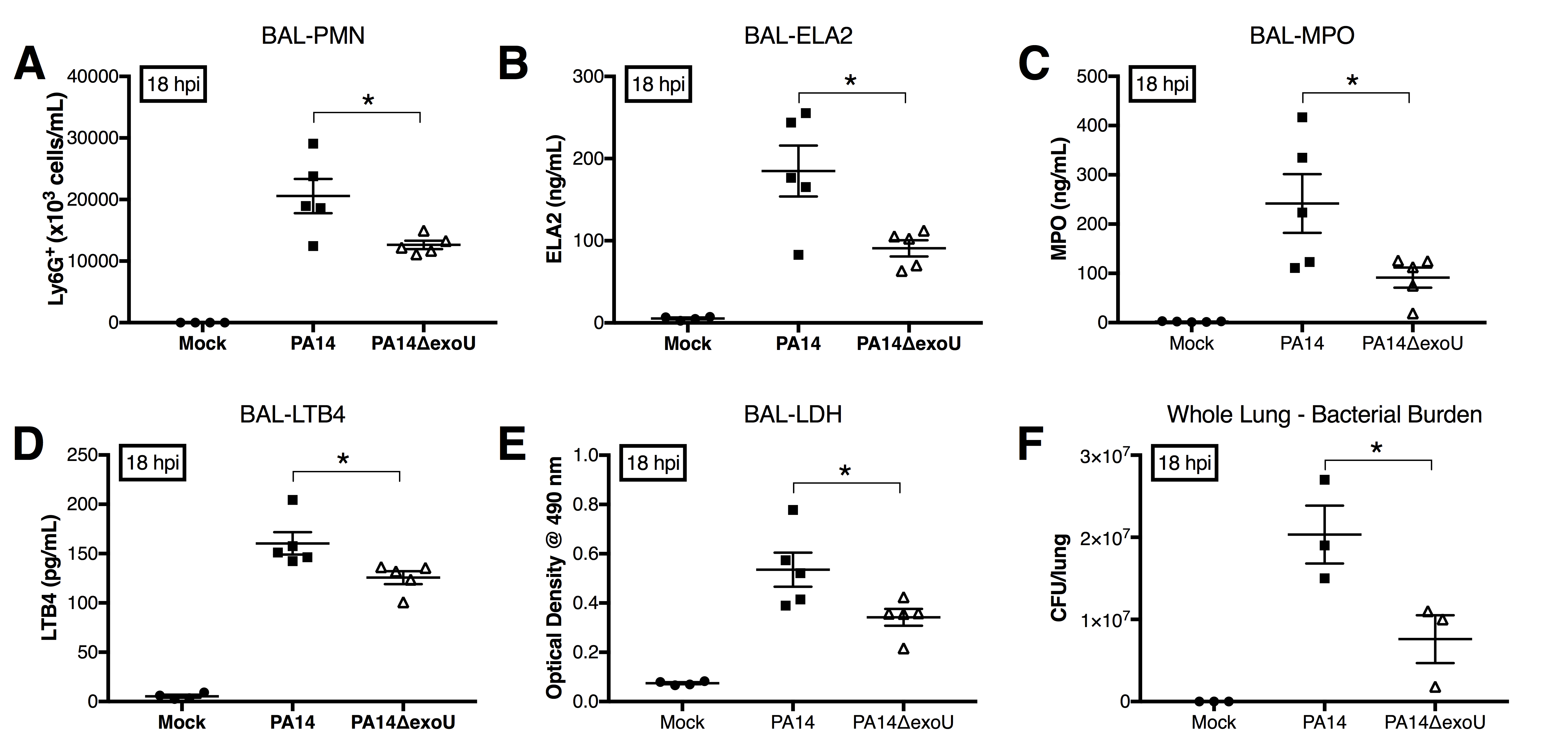

Supplement: S8 Fig — Adult 6–8 week old female C57BL/6J mice were challenged by intranasal inoculation of either PA14 or PA14ΔexoU. Bronchial alveolar lavage (BAL) samples were collected at 18h and total cell counts were performed. (A) Total cells were stained for Ly6G and analyzed by flow cytometry. Protein extracts were prepared from BAL samples and analyzed for (B) neutrophil elastase/ELA2 and (C) myeloperoxidase/MPO. (D) BAL cells were pelleted and supernatant liquids were tested for levels of LTB4 via ELISA. (E) LDH levels were assessed in the cell-free supernatant to assess cytotoxicity. (F) In independent experiments, 6–8 week old C57BL/6J female mice were infected and lung tissues were harvested 18hpi post infection to assess bacterial burden. Data are representative of at least four animals per group and are shown as mean +/- SEM, and are representative of multiple independent experiments. *p < 0.05. (TIFF) [file ppat.1006548.s008.tiff]
